# Supplementary material for: Detecting Genetic Mosaicism in Cultures of Human Pluripotent Stem Cells
Source: Stem Cell Reports. 2016 Nov 8;7(5):998–1012. doi: 10.1016/j.stemcr.2016.10.003 (PMC5106530; doi:10.1016/j.stemcr.2016.10.003)
Supplement: Document S1. Supplemental Experimental Procedures, Figures S1–S5, and Tables S1 and S2 [file mmc1.pdf]

Stem Cell Reports, Volume 7

Supplemental Information

Detecting Genetic Mosaicism in Cultures of Human Pluripotent Stem Cells

Duncan Baker, Adam J. Hirst, Paul J. Gokhale, Miguel A. Juarez, Steve Williams, Mark Wheeler, Kerry Bean, Thomas F. Allison, Harry D. Moore, Peter W. Andrews, and Ivana Barbaric

Supplementary Figure S1.

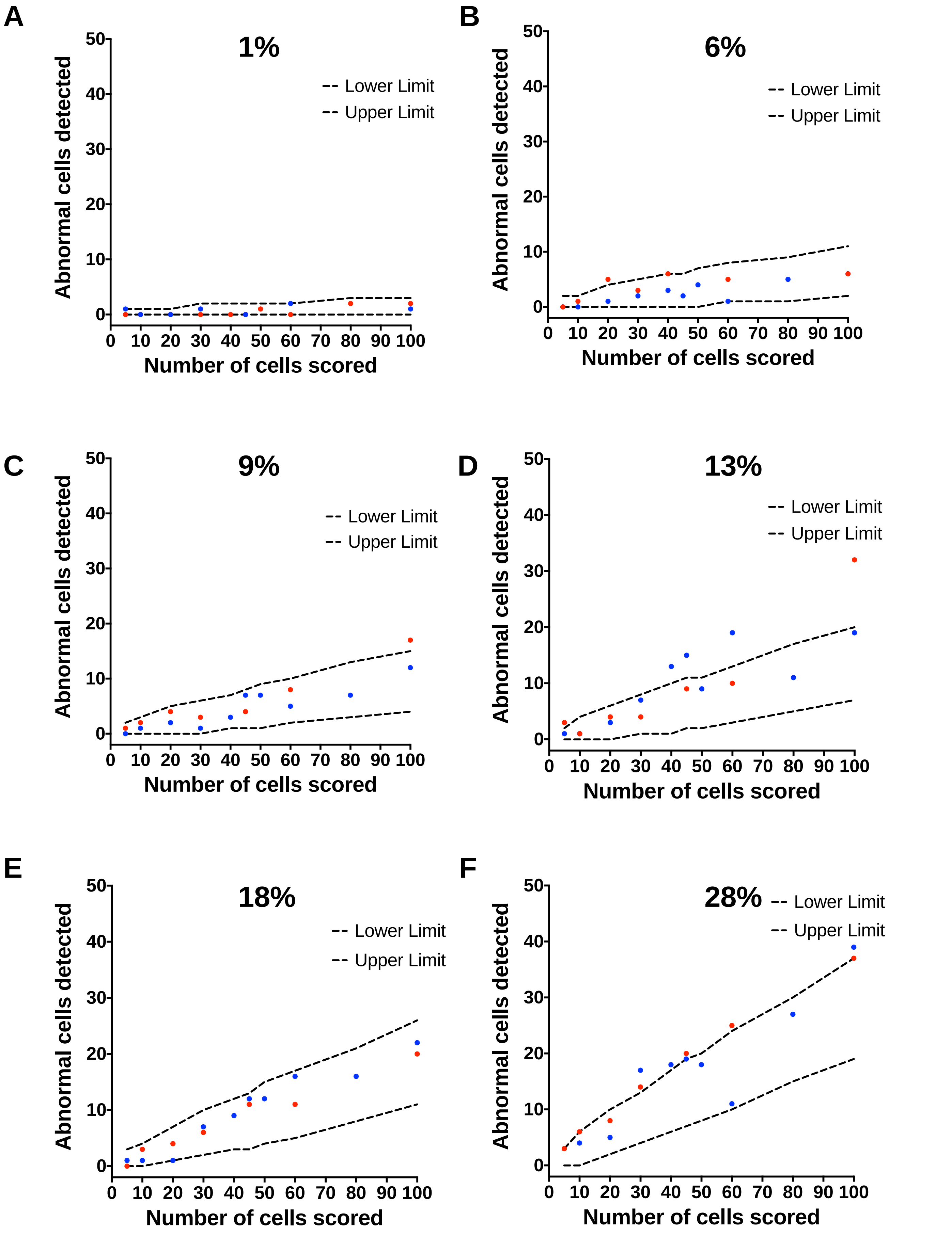

Supplementary Figure S2.

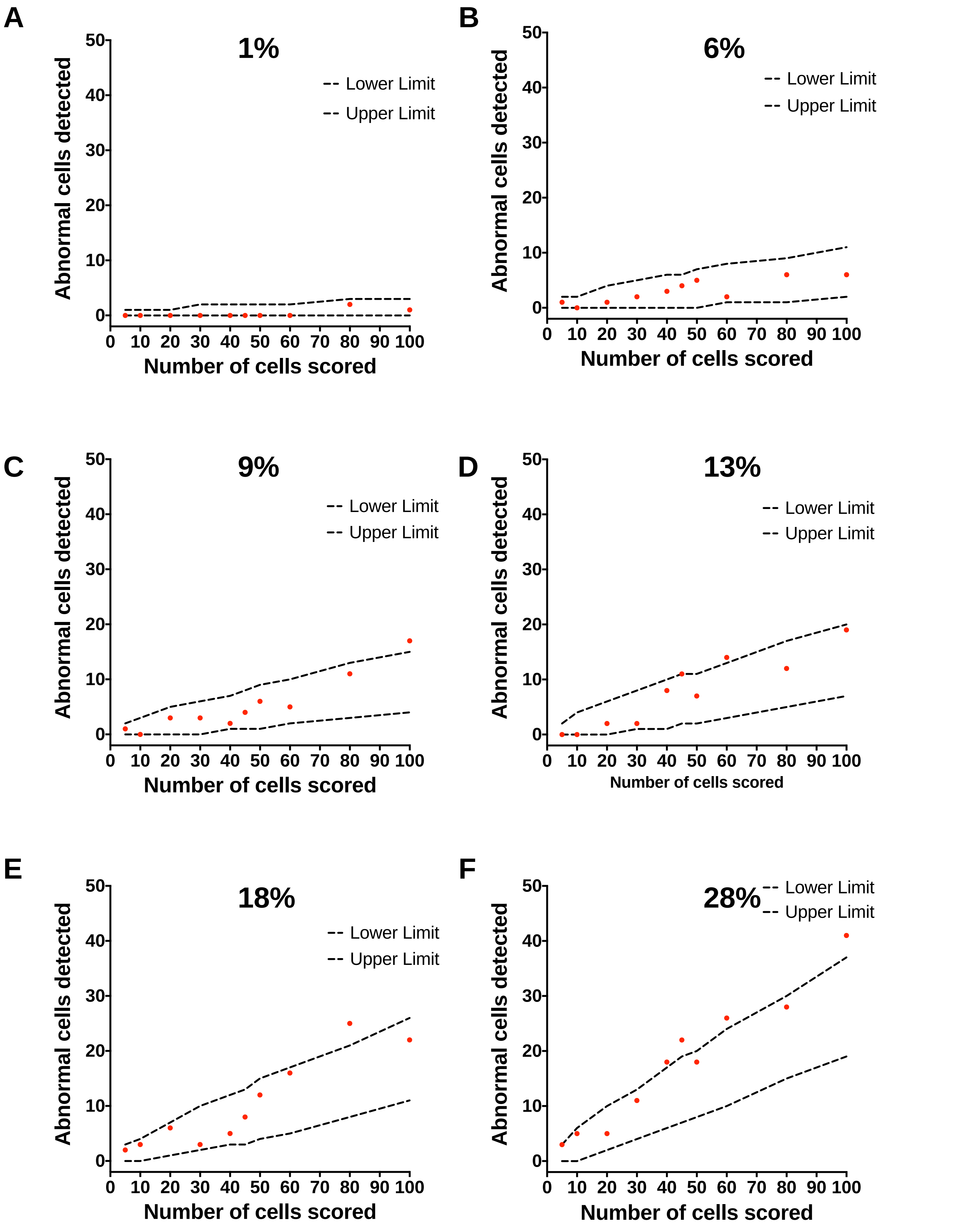

Supplementary Figure S3.

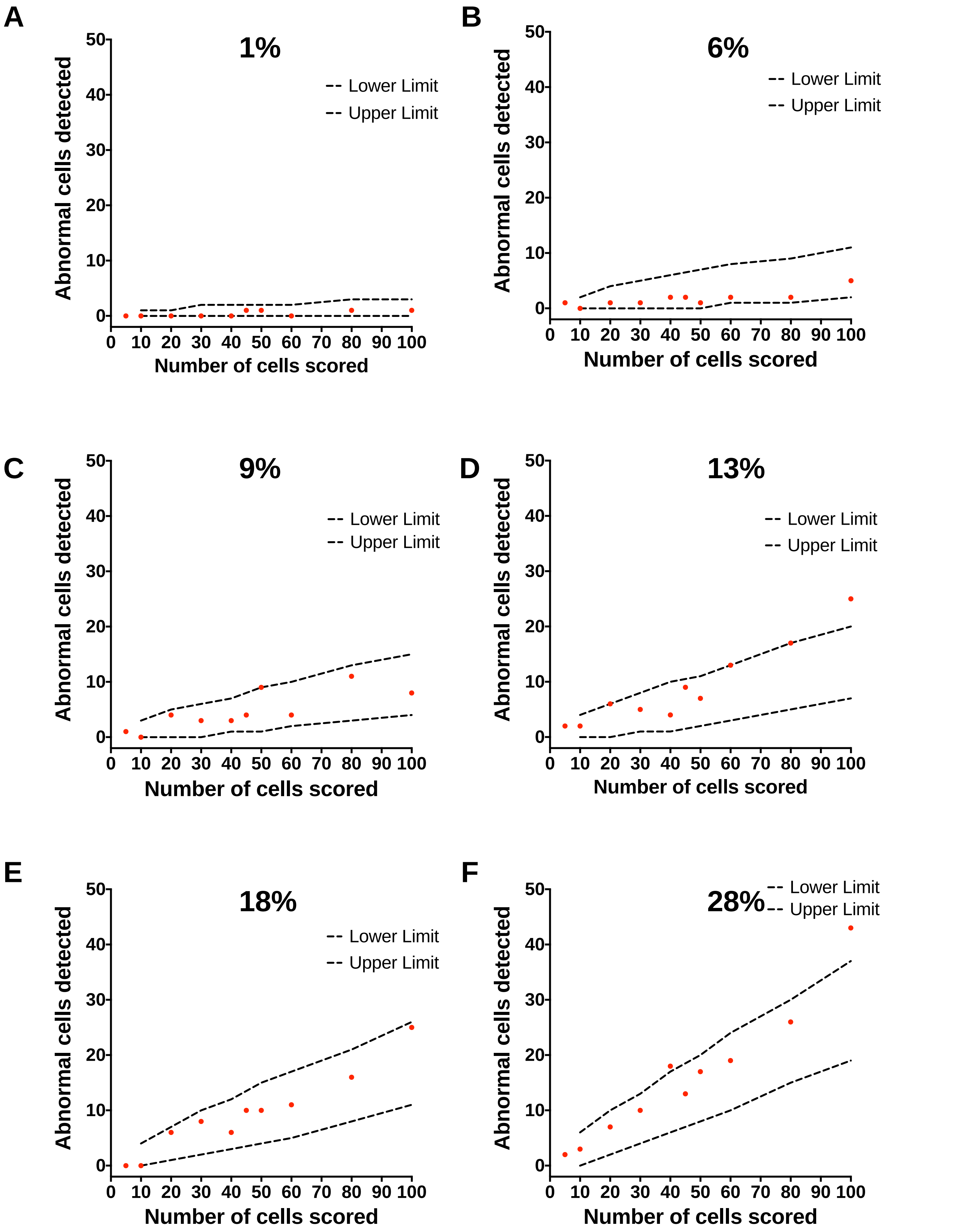

Supplementary Figure S4.

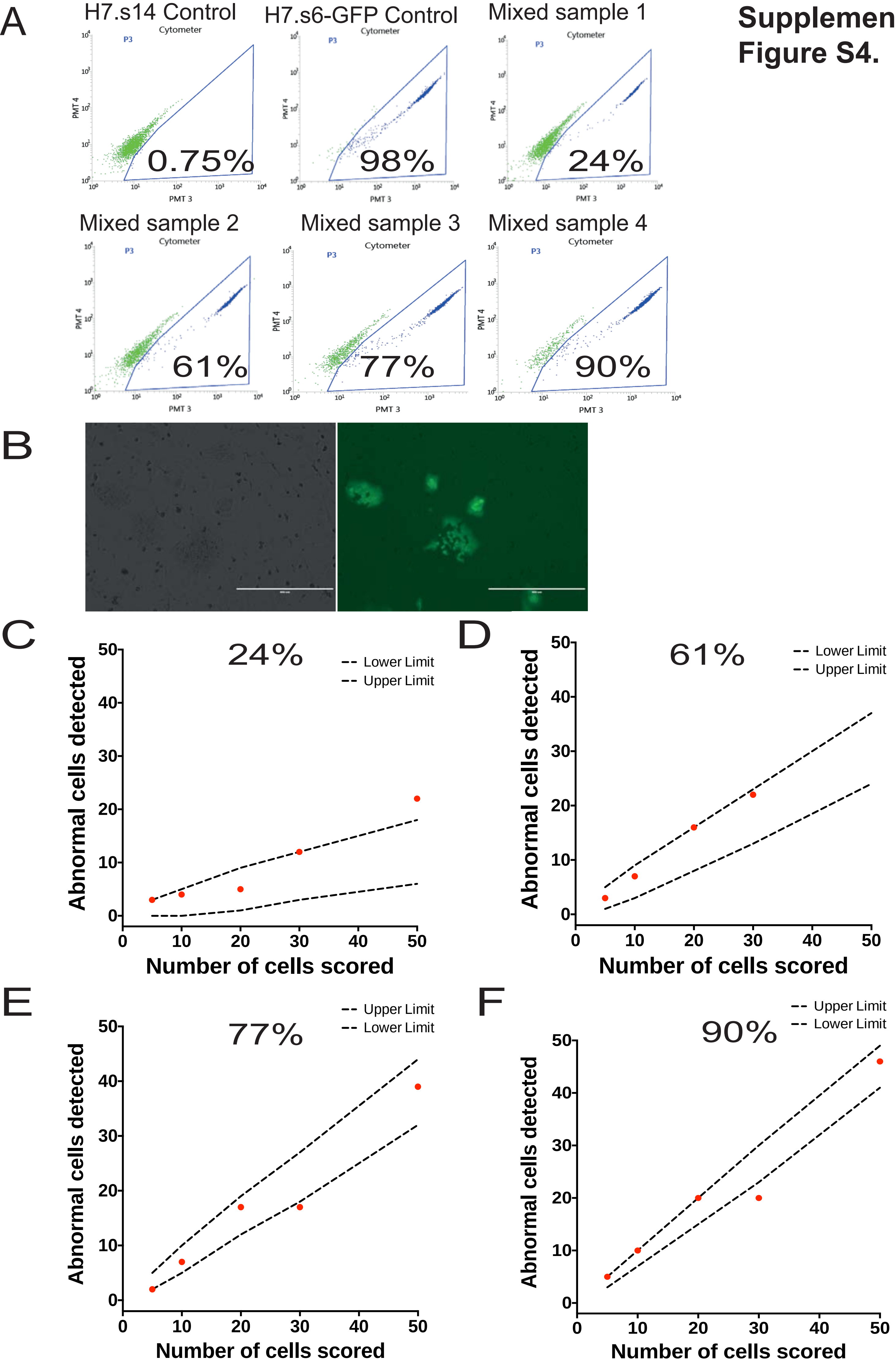

Supplementary Figure S5.

A

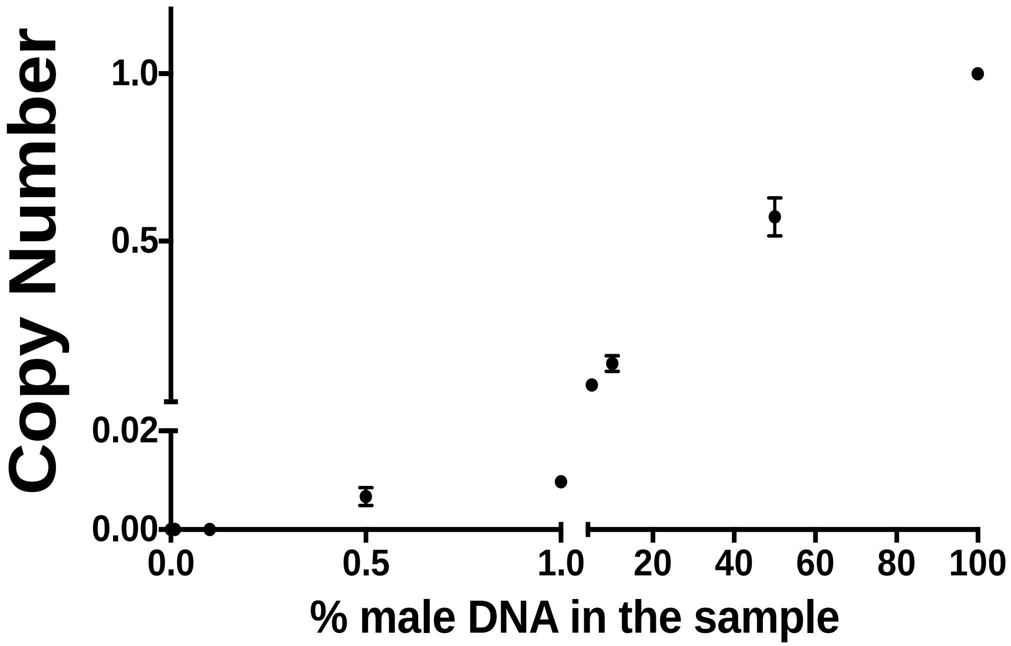

B

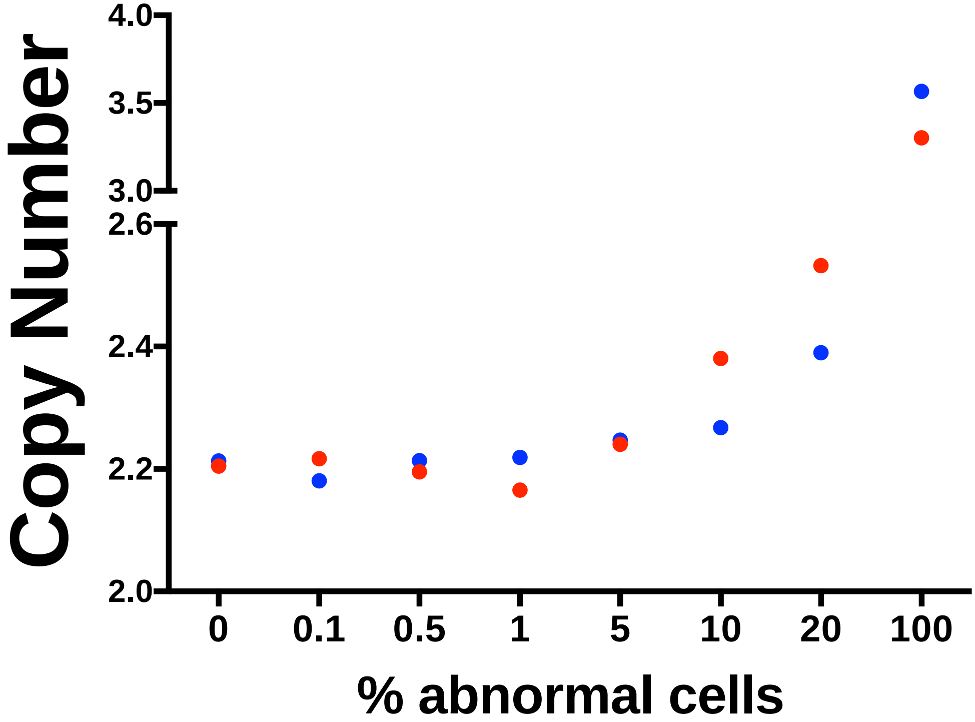

SUPPLEMENTAL INFORMATION

Supplemental Figure Legends

Figure S1. **The sensitivity of karyotyping in detecting mosaicism in hPSC cultures: analysis of the samples by a second clinical cytogeneticist; related to Figure 2.** Assessment of karyotyping sensitivity was performed using the same prepared slides of mosaic samples as in Figure 1 but the analysis was performed by a different cytogeneticist. Mixed samples containing (A) 1%, (B) 6%, (C) 9%, (D) 13%, (E) 18% and (F) 28% aneuploid cells (H7.s6-GFP) within the diploid cell population (H7.s14) were scored for the presence or absence of the abnormal chromosome 6, der(6)t(6;17)(q27;q1). Increasing numbers of metaphases (from five to 100) were scored from each sample. The numbers of abnormal cells detected in the duplicate analysis (red and blue circles) were plotted against statistically-determined expected numbers of abnormal cells (dotted lines) as detailed in the Supplementary Table S1.

Figure S2. The sensitivity of karyotyping in detecting mosaicism in hPSC cultures: analysis of new slides; related to Figure 2. New slides were prepared from the same mixed samples as analysed in Figures 1 and S1. Mixed samples containing (A) 1%, (B) 6%, (C) 9%, (D) 13%, (E) 18% and (F) 28% aneuploid cells (H7.s6-GFP) within the diploid cell population (H7.s14) were scored for the presence or absence of the abnormal chromosome 6, der(6)t(6;17)(q27;q1). Increasing numbers of metaphases (from five to 100) were scored from each sample. The numbers of abnormal cells detected (red circles) were plotted against statistically-determined expected numbers of abnormal cells (dotted lines) as detailed in the Supplementary Table S1.

Figure S3. The sensitivity of karyotyping in detecting mosaicism in hPSC cultures: a repeat of the experiment using fluorescently labelled H7.s14-Tomato subline; related to Figure 2. Diploid cells (H7.s14-Tomato, stably expressing tdTomato) and their aneuploid counterparts (H7.s6-GFP, stably expressing GFP) were mixed at different ratios (1-28%). The percentage of aneuploid cells was confirmed by imaging the cells on a high-content microscopy platform and using an associated image analysis software to count the number of GFP-expressing cells (H7.s6-GFP) within the total number of cells in the culture (total number of tdTomato-expressing and GFP-expressing cells). Mixed samples containing (A) 1%, (B) 6%, (C) 9%, (D) 13%, (E) 18% and (F) 28% aneuploid cells (H7.s6-GFP) within the diploid cell population (H7.s14-Tomato) were scored for the presence or absence of the abnormal chromosome 6, der(6)t(6;17)(q27;q1). Increasing numbers of metaphases (from five to 100) were scored from each sample. The numbers of abnormal cells detected in the duplicate analysis (red and blue circles) were plotted against statistically-determined expected numbers of abnormal cells (dotted lines) as detailed in the Supplementary Table S1.

Figure S4. The sensitivity of karyotyping in detecting mosaicism in hPSC cultures: a repeat of the experiment using a different way of mixing and counting cells; related to Figures 2 and 3. Diploid (H7.s14) and aneuploid (H7.s6-GFP, stably expressing GFP) cells were dissociated into single cells and mixed at different ratios. Mixed cultures were passaged once into two parallel flasks. (A) Flow cytometry was used to determine the ratio of abnormal cells in mixed cultures based on their GFP expression. The H7.s14 cells only (left panel) were used to set the gate and H7.s6-GFP cells only were used as a control. (B) Representative images of mixed cultures of H7.s14 diploid and H7.s6-GFP aneuploid cells containing 24% abnormal cells. Scale bar represents 400μm. Mixed samples containing (C) 24%, (D) 61%, (E) 77% and (F) 90% aneuploid cells (H7.s6-GFP) within the diploid cell population (H7.s14)

were scored for the presence or absence of the abnormal chromosome 6, der(6)t(6;17)(q27;q1). Increasing numbers of metaphases (from five to 50) were scored from each sample. The numbers of abnormal cells detected (red circles) were plotted against statistically-determined expected numbers of abnormal cells (dotted lines) as detailed in the Supplementary Table S1.

Figure S5. The sensitivity of the PCR-based assays in detecting mosaicism; related to Figures 5 and 6. (A) The sensitivity of qPCR for detecting copy number changes from zero to one. Plotted values on each graph are copy number means of technical triplicates calculated for each sample relative to the 0% control ± SEM. Related to Figures 5 and 6. (B) The sensitivity of the ddPCR assay on the RainDrop digital PCR platform. The ddPCR analysis of samples with varying ratios of abnormal cells harbouring 17q trisomy. Plotted are copy number values for two biological replicates.

Supplemental Tables

Table S1. Statistically-determined expected numbers of abnormal cells for different sample sizes (n) at given levels of mosaicism; related to Figures 2, 3, S1-S4. Each row represents a level of mosaicism (1-90%). The columns have lower (ll) and upper (ul) limits on the expected number of cells detected at 95% confidence for different sample sizes (n).

	**n=5**		**n=10**		**n=20**		**n=30**		**n=40**		**n=45**		**n=50**		**n=60**		**n=80**		**n=100**	
	**ll**	**ul**	**ll**	**ul**	**ll**	**ul**	**ll**	**ul**	**ll**	**ul**	**ll**	**ul**	**ll**	**ul**	**ll**	**ul**	**ll**	**ul**	**ll**	**ul**
**1%**	0	1	0	1	0	1	0	2	0	2	0	2	0	2	0	2	0	3	0	3
**2%**	0	1	0	1	0	2	0	2	0	3	0	3	0	3	0	4	0	4	0	5
**6%**	0	2	0	2	0	4	0	5	0	6	0	6	0	7	1	8	1	9	2	11
**9%**	0	2	0	3	0	5	0	6	1	7	1	8	1	9	2	10	3	13	4	15
**10%**	0	2	0	3	0	5	0	7	1	8	1	9	1	9	2	11	3	14	5	16
**13%**	0	2	0	4	0	6	1	8	1	10	2	11	2	11	3	13	5	17	7	20
**18%**	0	3	0	4	1	7	2	10	3	12	3	13	4	15	5	17	8	21	11	26
**19%**	0	3	0	5	1	7	2	10	3	13	4	14	4	15	6	18	9	22	12	27
**24%**	0	3	0	5	1	9	3	12	5	15	5	17	6	18	8	21	12	27	16	33
**28%**	0	3	0	6	2	10	4	13	6	17	7	19	8	20	10	24	15	30	19	37
**61%**	1	5	3	9	8	16	13	23	18	30	21	34	24	37	29	44	40	57	51	70
**77%**	2	5	5	10	12	19	18	27	25	36	29	40	32	44	40	52	54	69	69	85
**90%**	3	5	7	10	15	20	23	30	32	39	36	44	41	49	49	58	66	77	84	95

Table S2. Karyotypes of the cells used in the qPCR assays; related to Figure 4. For some of the cell lines the FISH analysis with a gene-specific DNA probe for the *BCL2L1* gene was performed in parallel with the qPCR and G-banding.

**HPSC line**	**Passage number**	**Karyotype [number of metaphases scored]**	**% cells with three copies of 20q11.21 as determined by FISH**
H7.s14	2+4+4+3+14	46,XX[30]	4%
Shef6 8H12	14+43+5+3+3+5	46,XX[30]	1%
Shef5	12+14+5+4+4	46,XX[20]	Not tested
MasterShef 8	21	46,XY[20]	8%
MasterShef 14	16	46,XX[30]	8%
H14.s9	9+6	46,XY[30]	Not tested
H7.s14-Tomato	23+3+10+6	46,XX[30]	14%
H14BJ1	92+11+6+5+6	48,XY,+12,+17,der(17)del(17)(p13)hsr(17)(p11.2)[10]	Not tested
Shef5-SF9	22+15+3+30+3+5+7	47,XX,t(1;11),trp(17)(p11.2),+20[20]	100%
H7.s6	62+33+2+4+34+5	47,XX,+del(1)(p22p22),der(6)t(6;17)(q27;q1)[10]	Not tested
HES3-MIXL	50+8/27+7+4+5+7	46,XX,add(10)(q24)[2]/46,XX[28]	53%
Shef6 2A7	14+43+5+3+3+6	46,XX[30]	41%

SUPPLEMENTAL EXPERIMENTAL PROCEDURES

The human pluripotent stem cell lines

H7 and H14 lines (Thomson et al. (1998)) were a gift from Dr James Thomson, University of Wisconsin. H7.s14, H7.s6, H14.s9 and H14BJ1 sublines were established at the University of Sheffield (Draper et al. (2004), Enver et al. (2005)). H7.s14-Tomato subline is stably expressing tdTomato fluorescent protein, and H7.s6-GFP and H14BJ1-GFP are stably expressing green fluorescent protein (GFP). MasterShef 8 and MasterShef 14 hPSC lines were derived to Good Manufacturing Practice Conditions at the University of Sheffield. Shef5 was derived at the University of Sheffield (Aflatoonian et al., 2010), and Shef5-SF9 subline was established at the University of Sheffield from the original Shef5 line. Shef6 8H12 and Shef6 2A7 are sublines of Shef6 hPSC line (Aflatoonian et al., 2010) derived at the University of Sheffield. HES3-MIXL is a HES3 line with a GFP knocked into the *MIXL1* allele obtained from A. Elefanty and E. Stanley (Davis et al., 2008).

Mixing experiments for detecting the sensitivity of karyotyping

H7.s14 or H7.s14-Tomato cells grown in mTESR medium (STEMCELL Technologies, Vancouver, Canada) on matrigel (BD Biosciences, Oxford, UK) were treated with collagenase IV, washed in mTESR and scraped off the flask to obtain small clumps of cells. The clumps were plated into 6 well plates (Corning Costar, High Wycombe, UK) on matrigel in mTESR and left for 48h. Cells from a spare well were counted to determine the cell numbers per well. H7.s6-GFP cells grown on matrigel in mTESR were dissociated into single cells using TrypLE (Life Technologies). Dissociated cells were washed with mTESR and counted. This was followed by plating of increasing numbers of dissociated H7.s6-GFP cells onto H7.s14 or H7.s14-Tomato cells grown in colonies to achieve the following ratios of

H7.s6-GFP cells in the H7.s14 or H7.s14-Tomato cultures: 1%, 6%, 9%, 13%, 18% and 28%. Mixed cultures were left for 24h to ensure that plated cells recover and restart cycling. The mixed cultures of H7.s14 and H7.s6-GFP cells were then stained with Hoechst 33342 and imaged on the InCell Analyser to confirm the ratios of normal and variant cells. The images were analysed using the Developer Toolbox 1.7 software (GE Healthcare) to determine the number of GFP expressing cells and the total number of nuclei based on Hoechst33342 staining. The ratios of abnormal cells were calculated as the number of GFP-expressing cells divided by the total number of nuclei per well. In the case of H7.s14-Tomato and H7.s6-GFP cells, the mixed cultures were imaged on the InCell Analyser for Tomato and GFP expression (without staining for Hoechst 33342) to confirm the ratios of cells plated. The images were analysed using the Developer Toolbox 1.7 software (GE Healthcare) to determine the number of GFP and tdTomato- expressing cells. The ratios of abnormal cells were determined as the number of GFP-expressing cells divided by the sum of the GFP-expressing and tdTomato-expressing cells. Following the imaging, mosaic cultures were treated with colcemid and processed for G-banding, as detailed below.

As an alternative way of constructing mosaic samples and determining the ratios of abnormal cells present in cultures, diploid cells (H7.s14 or H14.s9) and their GFP-expressing abnormal counterparts (H7.s6-GFP or H14BJ1-GFP) were dissociated into single cells using Single Cell Dissociation solution (Sigma-Aldrich, Poole, Dorset, UK) and counted. Different ratios of diploid and aneuploid cells were plated into flasks on vitronectin in Essential 8 and left to grow for 4-5 days. The flasks were then passaged at 1:2 ratio using ReLeSR (STEMCELL Technologies) and scraping, ensuring that all the cells are harvested from the flask. Each sample was passaged into two flasks on vitronectin in Essential 8. For H14 mixtures, the 10μM Y-27632 inhibitor (Sigma-Aldrich) was included in the media at the time of plating for 24h to aid with the survival of diploid cells. Two days post-plating, the cells

from one of the flasks were harvested using trypsin, ensuring all the cells are collected from the flask. After washing the cells in PBS supplemented with 10% fetal calf serum, the samples were analysed for the presence of GFP-labeled variant cells by flow cytometry using BD FACSJazz (BD Biosciences, Oxford, UK). H7.s14 or H14.s9 control samples were used to determine the gating threshold for GFP fluorescence and H7.s6-GFP or H14BJ1-GFP cells were used as a positive control for gating. At the same time, the duplicate flask was colcemid-treated and processed for G-banding, as detailed below. After the preparation of metaphase spreads on glass microscope slides, slides were scanned, metaphase images captured and analysed using the Leica Biosystems Cytovision Image Analysis system (version 7.3.2 build 35). The metaphase cells, having been thoroughly mixed in suspension during harvesting and slide making, were sorted based upon the location on the slide, achieving a random selection of cells for scoring. Metaphase cells were scored for the presence or absence of the abnormal chromosome 6, der(6)t(6;17)(q27;q1) in the H7.s14 and H7.s6-GFP mixed samples, or for the presence or absence of an additional abnormal chromosome 17, der(17)(del(17)(p13.3)hsr(17)(p11.2) in the H14.s9 and H14.BJ-GFP mixed samples. The standard number of metaphase cells used for a mosaic screen in clinical cytogenetics ranges from 30-45 (ACGS Best Practice Guidelines, http://www.acgs.uk.com) and therefore the number of cells scored here ranged from 5 to 100. Each metaphase cell was scored once only. Those cells in which the region of interest was unscorable (those where the chromosome was obscured, broken or missing) were excluded from analysis.

G-banding

Cells were treated with 0.1 µg/ml colcemid (Invitrogen) for up to 4h. After dissociation with 0.25% trypsin/versene (Gibco, Invitrogen), the cells were re-suspended in pre-warmed 0.0375M KCl hypotonic solution and incubated for 10min at room temperature. Cells were

then pelleted and resuspended in fixative (3:1 methanol:acetic acid). Metaphase spreads were prepared on glass microscope slides and G-banded by brief exposure to trypsin and stained with 4:1 Gurr's/Leishmann's stain (Sigma). Slides were scanned, metaphase images captured and analysed using the Leica Biosystems Cytovision Image Analysis system (version 7.3.2 build 35).

Statistically-determined expected numbers of abnormal cells for different sample sizes at given levels of mosaicism

A custom R code was implemented to calculate the upper and lower limits of the numbers of abnormal cells that would be expected when sampling a given number of cells, based on the confidence levels indicated in Hook et al. (1977).

DNA extraction and restriction digest

DNA from hPSCs was extracted using the Blood and Tissue Kit for DNA Extraction (QIAGEN, Crawley, UK). DNA was quantified by absorption spectroscopy (NanoPhotometer, Implen, Munich, Germany). Restriction digestion of DNA was performed in 100µl reactions containing 1µg of DNA and 10 units of *EcoRI* enzyme (Fermentas, ThermoFisher Scientific) in 1X *EcoRI* buffer (Fermentas). Reactions were incubated for 2h at 37°C and the enzyme was then inactivated by incubating at 65°C for 20min.

Primer design and validation

The genomic sequences of genes were obtained from the Ensembl Genome Browser Database. Gene-specific primers and the appropriate hydrolysis probes were designed in the intronic region of genes by the Universal Probe Library (UPL) Assay Design Centre web facility (Roche Diagnostics, Mannheim, Germany)

(http://lifescience.roche.com/shop/en/us/overviews/brand/universal-probe-library). NCBI Primer-BLAST was used to check primer specificity *in silico*. Newly designed primers were tested empirically using melting curve analysis. For this, 20µl PCR reactions were prepared in 384-well plates containing 1x SYBR Green JumpStart Taq Ready Master Mix (SigmaAldrich), 100nM of each of the forward and reverse primers, and 20ng of *EcoRI*-digested DNA template. Reactions were run on the QuantStudio 12K Flex Real-Time PCR System (Applied Biosystems, Life Technologies). Samples were heated to 50°C for 2min and denatured at 95°C for 10min. This was followed by 40 cycles of 95°C for 15s and 60°C for 1min. Melting curve analysis was performed by heating the samples to 95°C for 15s. The samples were then cooled and held at 60°C for 1min. This was followed by heating the samples to 95°C at a rate of 0.05°C/s under continuous fluorescence monitoring. The melting curves were obtained using the QuantStudio 12K Flex Software (Applied Biosystems, Life Technologies).

Primer amplification efficiencies for each set of primers were calculated by preparing a 5-fold dilution series of gDNA (100, 20, 4, 0.8 and 0.16ng) in triplicate or quadruplicate wells and plotting the average Cq values of replicates against the input DNA quantity in log10 scale. The data was fit to a straight line and the slope of the line was calculated. The primer efficiency was calculated as $-1+10^{(-1/slope)}$. Primers with efficiencies between 90% and 115% were used for the target and reference genes (**Table 1**).

Calculation of copy numbers of target genes relative to the calibrator samples in the qPCR assay

The Cq values obtained from the QuantStudio 12K Flex Software with auto baseline settings and were exported to Excel for copy number analysis using the relative quantification method (2^{-ddcq}) as follows:

[1] dCq(target gene) = Cq(target gene) – average Cq (reference gene, *RELL1*)

[2] ddCq1= dCq(target gene of the sample) – average dCq (target gene of the calibrator 1)

[3] Relative quantity of the target gene in the sample compared to calibrator 1 (RQ1) = 2^{-ddCq1}

[4] Copy number relative to calibrator 1 (CN1) = Number of copies of the target gene in the calibrator * RQ1 = 2* RQ1

[5] ddCq2= dCq(target gene of the sample) – average dCq (target gene of the calibrator 2)

[6] Relative quantity of the target gene in the sample compared to calibrator 2 (RQ2) = 2^{-ddCq2}

[7] Copy number relative to calibrator 2 (CN2) = Number of copies of the target gene in the calibrator * RQ2 = 2* RQ2

[8] Average copy number relative to both calibrators = Average of the CN1 and CN2

Digital droplet PCR using the RainDance PCR platform

Reaction mixtures (25µL) contained 1x TaqMan Genotyping Master Mix (ThermoFisher Scientific), 225nM of each primer and 50nM of each probe (TaqMan assays for *TK1* (FAM-labelled) and *TERT* (reference, VIC-labelled*)* (Life Technologies)), 1x dPCR Droplet Stabiliser (Raindance Technologies, Billerica, MA) and 150 ng DNA (as quantified by NanoPhotometer, Implen, Munich, Germany). Droplets were formed in the RainDrop source instrument (RainDance Technologies). The DNA was amplified in a thermal cycler using the following conditions: 95°C for 10min, followed by 45 cycles of 95°C for 15s and 60°C for 1min. The final extension step was at 98°C for 10min before the reactions were cooled down to 12°C and transferred into RainDrop Sense chip (RainDance Technologies) for fluorescence measurements of the probes (FAM and VIC). The data was analysed using the RainDrop Analyst Software (RainDance Technologies).

Supplemental Information for Figure 1:

Data of karyotypic abnormalities in human embryonic stem cells is based on the following references: (Amps et al., 2011; Baker et al., 2007; Brimble et al., 2004; Buzzard et al., 2004; Caisander et al., 2006; Catalina et al., 2008; Cowan et al., 2004; Draper et al., 2004; Gertow et al., 2007; Hasegawa et al., 2006; Herszfeld et al., 2006; Imreh et al., 2006; Inzunza et al., 2004; Lagarkova et al., 2008; Lefort et al., 2008; Ludwig et al., 2006; Maitra et al., 2005; Mitalipova et al., 2005; Narva et al., 2010; Spits et al., 2008; Ware et al., 2006).

Supplemental References

Aflatoonian, B., Ruban, L., Shamsuddin, S., Baker, D., Andrews, P., and Moore, H. (2010). Generation of Sheffield (Shef) human embryonic stem cell lines using a microdrop culture system. In Vitro Cell Dev Biol Anim *46*, 236-241.

Amps, K., Andrews, P.W., Anyfantis, G., Armstrong, L., Avery, S., Baharvand, H., Baker, J., Baker, D., Munoz, M.B., Beil, S.*, et al.* (2011). Screening ethnically diverse human embryonic stem cells identifies a chromosome 20 minimal amplicon conferring growth advantage. Nat Biotechnol *29*, 1132-1144.

Baker, D.E., Harrison, N.J., Maltby, E., Smith, K., Moore, H.D., Shaw, P.J., Heath, P.R., Holden, H., and Andrews, P.W. (2007). Adaptation to culture of human embryonic stem cells and oncogenesis in vivo. Nat Biotechnol *25*, 207-215.

Brimble, S.N., Zeng, X., Weiler, D.A., Luo, Y., Liu, Y., Lyons, I.G., Freed, W.J., Robins, A.J., Rao, M.S., and Schulz, T.C. (2004). Karyotypic stability, genotyping, differentiation, feeder-free maintenance, and gene expression sampling in three human embryonic stem cell lines derived prior to August 9, 2001. Stem Cells Dev *13*, 585-597.

Buzzard, J.J., Gough, N.M., Crook, J.M., and Colman, A. (2004). Karyotype of human ES cells during extended culture. Nat Biotechnol *22*, 381-382; author reply 382.

Caisander, G., Park, H., Frej, K., Lindqvist, J., Bergh, C., Lundin, K., and Hanson, C. (2006). Chromosomal integrity maintained in five human embryonic stem cell lines after prolonged in vitro culture. Chromosome Res *14*, 131-137.

Catalina, P., Montes, R., Ligero, G., Sanchez, L., de la Cueva, T., Bueno, C., Leone, P.E., and Menendez, P. (2008). Human ESCs predisposition to karyotypic instability: Is a matter of culture adaptation or differential vulnerability among hESC lines due to inherent properties? Mol Cancer *7*, 76.

Cowan, C.A., Klimanskaya, I., McMahon, J., Atienza, J., Witmyer, J., Zucker, J.P., Wang, S., Morton, C.C., McMahon, A.P., Powers, D.*, et al.* (2004). Derivation of embryonic stem-cell lines from human blastocysts. N Engl J Med *350*, 1353-1356.

Davis, R.P., Ng, E.S., Costa, M., Mossman, A.K., Sourris, K., Elefanty, A.G., and Stanley, E.G. (2008). Targeting a GFP reporter gene to the MIXL1 locus of human embryonic stem cells identifies human primitive streak-like cells and enables isolation of primitive hematopoietic precursors. Blood *111*, 1876-1884.

Draper, J.S., Smith, K., Gokhale, P., Moore, H.D., Maltby, E., Johnson, J., Meisner, L., Zwaka, T.P., Thomson, J.A., and Andrews, P.W. (2004). Recurrent gain of chromosomes 17q and 12 in cultured human embryonic stem cells. Nat Biotechnol *22*, 53-54.

Gertow, K., Cedervall, J., Unger, C., Szoke, K., Blennow, E., Imreh, M.P., and Ahrlund-Richter, L. (2007). Trisomy 12 in HESC leads to no selective in vivo growth advantage in teratomas, but induces an increased abundance of renal development. J Cell Biochem *100*, 1518-1525.

Hasegawa, K., Fujioka, T., Nakamura, Y., Nakatsuji, N., and Suemori, H. (2006). A method for the selection of human embryonic stem cell sublines with high replating efficiency after single-cell dissociation. Stem Cells *24*, 2649-2660.

Herszfeld, D., Wolvetang, E., Langton-Bunker, E., Chung, T.L., Filipczyk, A.A., Houssami, S., Jamshidi, P., Koh, K., Laslett, A.L., Michalska, A.*, et al.* (2006). CD30 is a survival factor and a biomarker for transformed human pluripotent stem cells. Nat Biotechnol *24*, 351-357.

Imreh, M.P., Gertow, K., Cedervall, J., Unger, C., Holmberg, K., Szoke, K., Csoregh, L., Fried, G., Dilber, S., Blennow, E.*, et al.* (2006). In vitro culture conditions favoring selection of chromosomal abnormalities in human ES cells. J Cell Biochem *99*, 508-516.

Inzunza, J., Sahlen, S., Holmberg, K., Stromberg, A.M., Teerijoki, H., Blennow, E., Hovatta, O., and Malmgren, H. (2004). Comparative genomic hybridization and karyotyping of human embryonic stem cells reveals the occurrence of an isodicentric X chromosome after long-term cultivation. Mol Hum Reprod *10*, 461-466.

Lagarkova, M.A., Volchkov, P.Y., Philonenko, E.S., Pfannkuche, K., Prokhorovich, M.A., Zabotina, T., Hescheler, J., and Kiselev, S.L. (2008). CD 30 is a marker of undifferentiated human embryonic stem cells rather than a biomarker of transformed hESCs. Cell Cycle *7*, 3610-3612.

Lefort, N., Feyeux, M., Bas, C., Feraud, O., Bennaceur-Griscelli, A., Tachdjian, G., Peschanski, M., and Perrier, A.L. (2008). Human embryonic stem cells reveal recurrent genomic instability at 20q11.21. Nat Biotechnol *26*, 1364-1366.

Ludwig, T.E., Levenstein, M.E., Jones, J.M., Berggren, W.T., Mitchen, E.R., Frane, J.L., Crandall, L.J., Daigh, C.A., Conard, K.R., Piekarczyk, M.S.*, et al.* (2006). Derivation of human embryonic stem cells in defined conditions. Nat Biotechnol *24*, 185-187.

Maitra, A., Arking, D.E., Shivapurkar, N., Ikeda, M., Stastny, V., Kassauei, K., Sui, G., Cutler, D.J., Liu, Y., Brimble, S.N.*, et al.* (2005). Genomic alterations in cultured human embryonic stem cells. Nat Genet *37*, 1099-1103.

Mitalipova, M.M., Rao, R.R., Hoyer, D.M., Johnson, J.A., Meisner, L.F., Jones, K.L., Dalton, S., and Stice, S.L. (2005). Preserving the genetic integrity of human embryonic stem cells. Nat Biotech *23*, 19-20.

Narva, E., Autio, R., Rahkonen, N., Kong, L., Harrison, N., Kitsberg, D., Borghese, L., Itskovitz-Eldor, J., Rasool, O., Dvorak, P.*, et al.* (2010). High-resolution DNA analysis of human embryonic stem cell lines reveals culture-induced copy number changes and loss of heterozygosity. Nat Biotechnol *28*, 371-377.

Spits, C., Mateizel, I., Geens, M., Mertzanidou, A., Staessen, C., Vandeskelde, Y., Van der Elst, J., Liebaers, I., and Sermon, K. (2008). Recurrent chromosomal abnormalities in human embryonic stem cells. Nat Biotechnol *26*, 1361-1363.

Ware, C.B., Nelson, A.M., and Blau, C.A. (2006). A comparison of NIH-approved human ESC lines. Stem Cells *24*, 2677-2684.
