# Supplementary material for: Detecting Genetic Mosaicism in Cultures of Human Pluripotent Stem Cells
Source: Stem Cell Reports. 2016 Nov 8;7(5):998–1012. doi: 10.1016/j.stemcr.2016.10.003 (PMC5106530; doi:10.1016/j.stemcr.2016.10.003)
Supplement: Document S2. Article plus Supplemental Information [file mmc2.pdf]

Stem Cell Reports
Resource
OPEN ACCESS

Detecting Genetic Mosaicism in Cultures of Human Pluripotent Stem Cells

Duncan Baker,[1,2] Adam J. Hirst,[1] Paul J. Gokhale,[1] Miguel A. Juarez,[3] Steve Williams,[2] Mark Wheeler,[2] Kerry Bean,[2] Thomas F. Allison,[1] Harry D. Moore,[1] Peter W. Andrews,[1] and Ivana Barbaric[1,*]
[1]Department of Biomedical Science, Centre for Stem Cell Biology, The University of Sheffield, Sheffield S10 2TN, UK
[2]Sheffield Diagnostic Genetic Services, Sheffield Children's Hospital, Sheffield S10 2TH, UK
[3]School of Mathematics and Statistics, The University of Sheffield, Sheffield S3 7RH, UK
*Correspondence: i.barbaric@sheffield.ac.uk
http://dx.doi.org/10.1016/j.stemcr.2016.10.003

SUMMARY

Genetic changes in human pluripotent stem cells (hPSCs) gained during culture can confound experimental results and potentially jeopardize the outcome of clinical therapies. Particularly common changes in hPSCs are trisomies of chromosomes 1, 12, 17, and 20. Thus, hPSCs should be regularly screened for such aberrations. Although a number of methods are used to assess hPSC genotypes, there has been no systematic evaluation of the sensitivity of the commonly used techniques in detecting low-level mosaicism in hPSC cultures. We have performed mixing experiments to mimic the naturally occurring mosaicism and have assessed the sensitivity of chromosome banding, qPCR, fluorescence in situ hybridization, and digital droplet PCR in detecting variants. Our analysis highlights the limits of mosaicism detection by the commonly employed methods, a pivotal requirement for interpreting the genetic status of hPSCs and for setting standards for safe applications of hPSCs in regenerative medicine.

INTRODUCTION

The dual ability of human pluripotent stem cells (hPSCs) to self-renew and differentiate into any cell type in the body makes them a promising cell source for regenerative medicine, disease modeling, and drug discovery (Takahashi et al., 2007; Thomson et al., 1998). Such applications necessitate the maintenance of large numbers of undifferentiated, genetically stable cells. However, hPSCs are subject to genetic changes in vitro and in the presence of selection pressures, the variants with changes that allow for improved growth outcompete their neighbors and overtake the culture (Draper et al., 2004; Olariu et al., 2010). The commonly observed genetic changes in hPSCs are non-random and involve gains of either parts or whole chromosomes 1, 12, 17, and 20 (Amps et al., 2011; Taapken et al., 2011), indicating that genes within these regions confer selective advantage to variant cells (Avery et al., 2013; Blum et al., 2009).

Genetic aberrations that arise in hPSCs during culture can affect their behavior and confound experimental results. Some of the variant cells with common genetic changes show signs of neoplastic progression (Werbowetski-Ogilvie et al., 2009), including reduced apoptosis (Avery et al., 2013; Yang et al., 2008), growth-factor independence (Werbowetski-Ogilvie et al., 2009) and higher cloning efficiency (Barbaric et al., 2014; Enver et al., 2005). Genetic changes can also affect the differentiation propensity of hPSCs. For example, a culture-adapted H7 line displayed a reduced tendency for differentiation to endoderm (Fazeli et al., 2011). Similarly, variant cells with a gain of chromosome 20q11.1-11.2 showed differences in the hematopoietic and neural differentiation protocols compared with their wild-type controls (Werbowetski-Ogilvie et al., 2009). Altered patterns of differentiation caused by accrued genetic changes may significantly affect the use of such cell lines in applications that require the production of differentiated derivatives. Furthermore, the commonly observed genetic changes in hPSCs are also frequently observed in embryonal carcinoma cells, the stem cells of malignant germ cell tumors termed teratocarcinomas (Harrison et al., 2007). Indeed, gain of chromosome 12p is used as a diagnostic marker for testicular germ cell tumors (Sandberg et al., 1996). With hPSCs derivatives entering clinical trials, a possibility that genetic changes may confer malignant properties to hPSCs or their differentiated progeny is a cause of regulatory concern (Goldring et al., 2011). Consequently, scientists using hPSCs need to be vigilant to monitor the cultures for the presence of genetic changes. This necessitates a good understanding of the sensitivities of different methods used for screening hPSC cultures, as preparations of cells declared "normal" and "free of genetic variants" according to a particular methodology may nevertheless harbor variant cells below the level of sensitivity.

Traditionally, testing of hPSC lines for gross chromosomal changes employed karyology by chromosome banding of metaphase cells (Amps et al., 2011; Baker et al., 2007). Although karyotyping allows examination of the entire cell genome in a single assay, an often overlooked issue in evaluating hPSC cultures is the sensitivity of karyotyping in the detection of a low-grade mosaicism. Clinical cytogeneticists have an established set of criteria for the number of metaphases that need to be screened to detect the presence of variant cells in clinical samples with a certain level of confidence. For example, analysis of 30

 © 2016 The Authors.
This is an open access article under the CC BY license (http://creativecommons.org/licenses/by/4.0/).

metaphases excludes 10% mosaicism, whereas analysis of 50 metaphases excludes 6% mosaicism, both with 95% confidence (Hook, 1977). However, such calculations are based on a statistical random sampling of a homogeneous population and there is a question of whether this is applicable to hPSCs, given that they grow in adherent cultures as colonies and may not behave as a homogeneous population when dissociated.

Even with the appropriate numbers of metaphases sampled, karyotyping has additional shortfalls. The analysis is limited by the fact that only mitotic cells can be assessed, and it is also labor intense and relatively expensive. Furthermore, karyotyping has a limited resolution of about 5–10 Mb (Shaffer et al., 2013). Although there are occasions when abnormalities of less than 5 Mb could be detected, this is limited to very specific karyotype changes in which the chromosome band size, location (small, clear separation from neighboring bands), and staining intensity allows for such a small change to be visible. The limited resolution may present a problem for assessing the genetic status of hPSC cultures, as some of the most common changes in hPSCs are present at a subkaryotype level. Common structural variants are a gain of 20q11.21 copy-number variant (CNV) (Amps et al., 2011; Lefort et al., 2008; Martins-Taylor et al., 2011) or loss of 10p13-pter, 18q21-qter, and 22q13-qter (Amps et al., 2011). In the International Stem Cell Initiative study, 20q CNV was identified in more than 20% of the 120 lines analyzed, and 22 of the lines harboring 20q CNV appeared normal by karyology (Amps et al., 2011). Thus, detecting this particularly frequent change usually requires the use of alternative methods such as fluorescence in situ hybridization (FISH) on interphase cells. The use of such a labor-intensive and expensive method is limiting the frequency of assessing genetic stability of hPSCs during routine maintenance. Hence, there is a need for a rapid, cost-effective assay that could be employed in common laboratory practice for regular screening of hPSCs for common genetic changes.

qPCR offers a rapid alternative method to karyotyping or FISH for detecting copy-number changes (Hoebeeck et al., 2007). Unlike karyotyping, which provides a view of the whole genome of a cell, qPCR and FISH-based methods are target specific and hence typically serve as complementary analyses to genome-wide methods (Gekas et al., 2011; Olde Nordkamp et al., 2009). D'Hulst et al. (2013) have employed qPCR as a way of rapid detection of common karyotypic changes in murine PSCs. Their assays for a gain of chromosome 8 or a loss of chromosome Y were able to detect abnormal cells when they were present in 10% or more of cells in culture (D'Hulst et al., 2013). Similarly, Avery et al. (2013) have used a qPCR assay for detecting a gain of chromosome 20q11.21 in hPSCs. However, the limitations of this assay in respect of detecting low-level mosaicism in hPSCs remain unclear. More recently, digital PCR has been developed to allow absolute quantification of DNA and to afford higher sensitivity and precision in detecting mutant alleles or copy-number variation (Vogelstein and Kinzler, 1999).

Here, we first tested the assumption that karyotyping of hPSCs conforms to random sampling rules used in clinical cytogenetics. We found that the numbers of metaphases required to be scored match well the numbers anticipated from random sampling theory, and scoring abnormal variants was not significantly distorted by different growth characteristics of the variant cells. Furthermore, we report a qPCR assay as a rapid and accessible method for assessing frequently occurring genetic aberrations in hPSCs. Finally, our analyses show that although karyotyping, FISH, qPCR, and digital PCR are effective methods for monitoring the appearance of known common genetic variants, none of these methods reliably detect variants if they are present at less than approximately 5%–10% of the cells in a culture.

RESULTS

The Common Genetic Changes in hPSC Cultures

To analyze the spectrum of genetic changes in hPSCs, we have assembled the published data reporting genetic abnormalities in human embryonic stem cells (Figure 1). Although aberrations in all of the chromosomes were observed, the distribution of cytogenetic abnormalities appears non-random, as was previously reported (Amps et al., 2011). The most common alterations are the gains of whole chromosomes and duplications of chromosomal regions, representing 42% and 31% of all karyotypic abnormalities reported, respectively. The most common alteration detected by chromosomal banding is a gain of chromosome 17 (17% of all karyotypic abnormalities reported). Additional chromosomes that are frequently gained (detected by chromosomal banding) are chromosomes 12 (13.8% of all karyotypic abnormalities reported), 1 (8%), 20 (7.4%), and X (5.5%). All of these chromosomes have been reported at least twice as a sole abnormality in cells. However, unlike chromosomes 12 and X, chromosome 1 is rarely gained as a whole chromosome and the gains of chromosome 1 appear mostly as unbalanced structural rearrangements. The overlapping structural variants have been used to narrow down chromosomal regions on frequently gained chromosomes to aid in identifying genes that may be driving selective advantage of genetically variant cells. The minimal amplicons for 1, 12, 17, and 20 have been identified at 1q25-q41, 12p11-pter, 17q25-qter, and 20q11.21, respectively.

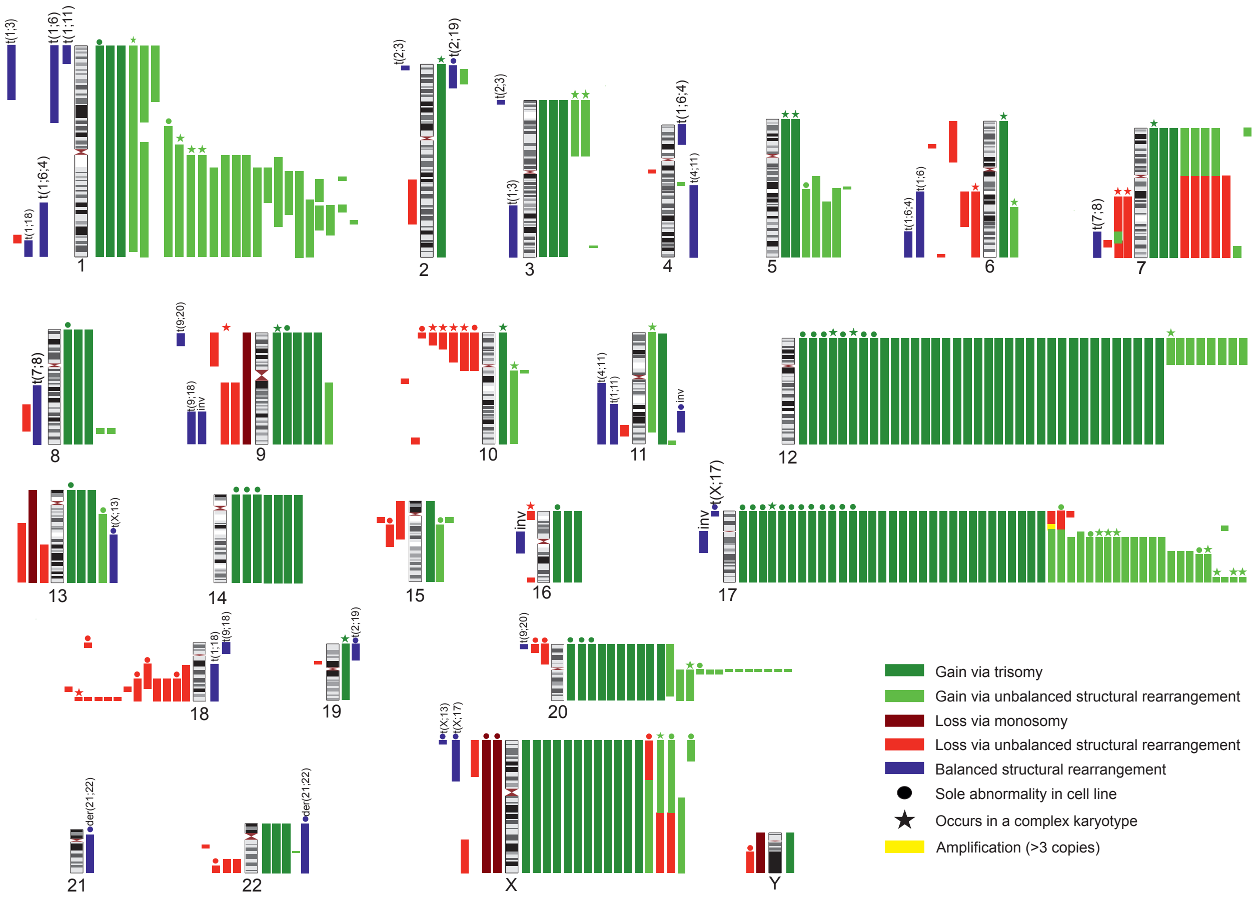

Figure 1. Ideogram Summarizing the Reported Chromosome Changes in Human Embryonic Stem Cell Lines
Each colored bar represents one chromosome change occurrence in one cell line. Chromosome losses and gains are shown to the left and right of the ideogram, respectively, except that in those instances where a single chromosome rearrangement results in a gain and a loss, the colored bars are shown together for clarity. The cytogenetic changes are color coded: maroon, loss of a whole chromosome (monosomy); red, loss via a structural chromosome rearrangement (unbalanced translocation or interstitial deletion); dark green, gain of a whole chromosome (trisomy); light green, gain via a structural chromosome rearrangement (unbalanced translocation or interstitial duplication); blue, occurrence of an apparently balanced rearrangement the nature of which is labeled. Instances in which a change affected only a single chromosome are denoted by a filled circle, whereas changes associated with complex karyotypes (>5 unrelated chromosome aberrations) are denoted by a star. The list of references used for this summary can be found in the Supplemental References.

Losses of chromosomes or chromosomal material occur less frequently than the gains (22% of all karyotypic abnormalities reported are losses versus 73% reported gains) and they occur predominantly as a loss of a chromosomal region (20% of reported changes) rather than monosomy (2% of reported changes). A loss of an entire chromosome was reported only for two of the somatic chromosomes (chromosomes 9 and 13), although monosomy of these chromosomes was not the only abnormality seen in the cells. The loss of X chromosome was reported twice as a sole abnormality. The minimal regions most commonly subject to loss are 10p13-pter and 18q21-qter. Chromosomes that are rarely seen as gained or lost via unbalanced rearrangements in hPSCs are chromosomes 4, 19, and 21 (Figure 1).

Sensitivity of Karyotyping in Detecting Mosaicism in hPSC Cultures

To assess the sensitivity of chromosome banding in detecting low levels of mosaicism in culture, we performed mixing experiments of diploid (H7.s14, 46,XX) and variant trisomic cells stably transfected with GFP (H7.s6-GFP, 48,XX,+del(1)(p22p22),der(6)t(6;17)(q27;q1),+12[19]/49,XX, idem,+20[1]). Samples with increasing ratios of variant cells in diploid cultures (from 1% to 28%, as assessed by high-content imaging of the numbers of

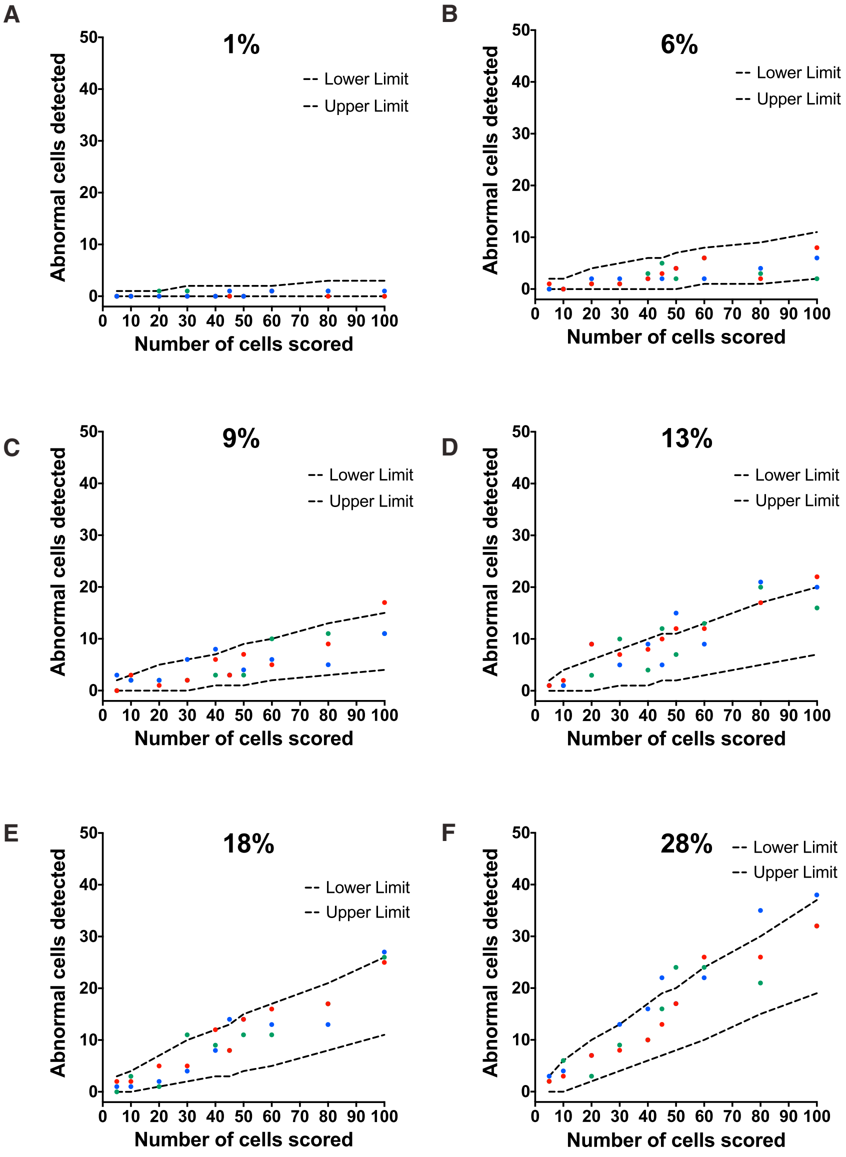

Figure 2. Sensitivity of Karyotyping in Detecting Mosaicism in hPSC Cultures

Diploid (H7.s14) and aneuploid (H7.s6-GFP, stably expressing GFP) cells were mixed at different ratios. The percentage of aneuploid cells was confirmed by imaging the cells on a high-content microscopy platform and using associated image analysis software to count the number of aneuploid cells based on GFP expression. Mosaic cultures containing (A) 1%, (B) 6%, (C) 9%, (D) 13%, (E) 18%, and (F) 28% aneuploid cells within the diploid cell population were subjected to karyotypic analysis. Metaphase cells were scored for the presence or absence of the abnormal chromosome 6, der(6)t(6;17)(q27;q1) in the mixed samples. Increasing numbers of metaphases (from 5 to 100) were scored from each sample. The numbers of abnormal cells detected in triplicate analysis (red, green, and blue circles) were plotted against statistically determined expected numbers of abnormal cells (dashed lines) as detailed in Table S1.

GFP-expressing cells) were treated with colcemid and processed for G-banding. Increasing numbers of unique metaphases (from 5 to 100) were then sampled from the slides containing the chromosomal spreads and chromosome numbers were counted in the cells (Figure 2). Overall, the numbers of abnormal cells detected in the samples fell within the expected confidence limits (Figure 2 and Table S1). However, at higher ratios of mosaicism (>13%), some of the numbers of abnormal cells detected were higher than the statistically predicted ones (Figures 2D–2F). To exclude a potential operator bias in detecting the numbers of abnormal cells, a second independent analyst scored the metaphases on the same cytogenetic slides and a similar trend in the number of abnormal cells was observed (Figure S1). New cytogenetic slides were also prepared from the existing samples and counted (Figure S2). A repeat of the experiment using tdTomato-labeled diploid line (H7.s14-Tomato, 46,XX) and the GFP-labeled variant line (H7.s6-GFP) also showed similar results, with the numbers of abnormal cells matching well the statistically predicted numbers (Figure S3).

As an alternative way of constructing mosaic samples and determining the ratios of abnormal cells present in cultures, we dissociated diploid cells (H7.s14) and their triploid counterparts (H7.s6-GFP) to single cells, counted them, and seeded them into flasks. The mosaic cultures were passaged once to further ensure inter-mixing of normal and variant cells within the cultures. At the time

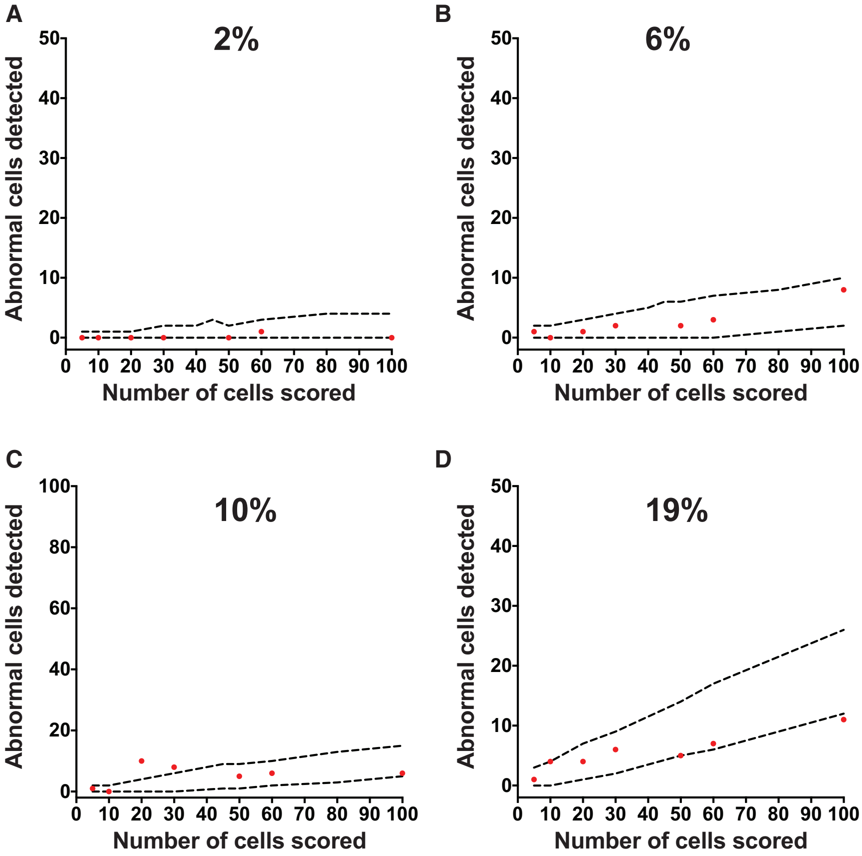

Figure 3. Sensitivity of Karyotyping in Detecting Mosaicism in hPSC Cultures Tested on a Different Pair of Normal and Genetically Variant Cells

Diploid (H14.s9) and aneuploid (H14BJ1-GFP) cells were mixed at different ratios. Flow cytometry was used to determine the ratio of abnormal cells in mixed cultures based on their GFP expression. Mosaic cultures containing (A) 2%, (B) 6%, (C) 10%, and (D) 19% aneuploid cells within the diploid cell population were subjected to karyotypic analysis. Metaphase cells were scored for the presence or absence of an additional abnormal chromosome 17, der(17) del(17)(p13.3)hsr(17)(p11.2). Increasing numbers of metaphases (from 5 to 100) were scored from each sample. The numbers of abnormal cells detected (red circles) were plotted against statistically determined expected numbers of abnormal cells (dotted lines) (Table S1).

of passage each sample was split into duplicate flasks. Two days after plating one of the flasks was used to determine the ratios of normal and variant cells by flow cytometry, whereas the duplicate flask was processed for G-banding. The mosaic samples contained between 24% and 90% abnormal cells (Figure S4A) which intermixed well with the diploid cells, creating "salt and pepper" type colonies (Figure S4B). Scoring of up to 50 metaphases from such mosaic cultures also matched well the numbers of statistically predicted abnormal cells (Figure S4C and Table S1).

Finally, we repeated the latter mixing experiment using a pair of normal and genetically variant sublines of the H14 hPSC line. The diploid (H14.s9 [46,XY]) and GFP-expressing variant cells (H14.BJ1-GFP [48,XY,+12,+17,der(17) del(17)(p13)hsr(17)(p11.2)[10]]) were dissociated to single cells, mixed at different ratios, and plated. Established cultures were passaged once into duplicate flasks and processed for G-banding or flow cytometry, as described above. Similar to our observations on H7 line, scoring of up to 100 metaphases from mixed cultures with different ratios of abnormal cells was in line with the statistically predicted numbers (Figure 3 and Table S1).

Overall, in repeated experiments the numbers of metaphases scored as abnormal matched well the numbers anticipated from random sampling theory, and scoring genetically abnormal variants was not substantially distorted by different growth characteristics of the variant cells. However, the low level of mosaicism (1%) could not be reliably detected even at 100 metaphases analyzed.

qPCR Assay for Detecting the Most Commonly Identified Genetic Changes in hPSCs

We next sought to design a qPCR assay that could be used as a fast and accessible method for detecting frequent genetic changes in hPSCs. Given that whole or partial trisomies 1, 12, 17, and 20 account for the majority of aneuploidies in hPSCs (Figure 1), we designed primers located on these chromosomes. Chromosome 4 is rarely gained or duplicated in hPSCs; therefore, we chose a gene on chromosome 4 (*RELL1*) as a reference gene. All primer pairs were designed to bind within the introns of selected genes. The predicted PCR amplicons were checked for the absence of *Eco*RI restriction site as the gDNA extracted from hPSCs was digested with *Eco*RI to enable accessibility of primers in PCR. Initially we designed multiple primer pairs for each of the chromosomal loci and checked their specificity using NCBI Primer-BLAST. To validate the primers empirically, we first used them in a qPCR reaction and compared

Table 1. Primers and Probes Used in qPCR

Gene	Gene Location	Primer Sequences (5′-3′)	UPL Probe	Amplicon Size (bp)	Primer Efficiency (%)
NPHP4	1p36	F: ccggcctatcgtcactttt<br>R: gccggtgtgtgcagaact	8	60	94
CHD1L	1q12	F: aaaaacctaagtaacagagggacatt<br>R: tgtatctatgttgttgggattcatact	56	69	101
RELL1	4p14	F: tgcttgctcagaaggagctt<br>R: tgggttcaggaacagagaca	12	64	94
DPPA3	12p13.31	F: cgtagcgtcgttgcatca<br>R: tcctttttaccgttcctgaca	60	63	96
LGR5	12q21.1	F: gatatgttggggattgacacg<br>R: tgctcaaagaggacaaccttc	6	60	109
FLCN	17p11.2	F: tgcagtccacaatgacaagtg<br>R: ccatgagagccgaagactgt	68	74	101
TK1	17q23.2-q25.3	F: ggtgacagctgcttacagcttag<br>R: actggttgccaccttctcag	60	64	101
BCL2L1	20q11.21	F: tctgcagaaggctaccccta<br>R: tgctgtgtctaagacctctttcat	44	75	111

Gene name and location, primer sequences, and Universal Probe Library (UPL) probes used for amplification of target sequences. F, forward primer; R, reverse primer.

the average values of the cycle threshold (Cq) of technical triplicates of the target gene to the reference gene on chromosome 4 (*RELL1*). Primer pairs with a Cq value within two units of the reference gene were tested for single product amplification and primer efficiency. To ensure single band amplification, we used primers in a qPCR reaction with a melting curve analysis. Primer pairs that produced a single amplicon were further tested for amplification efficiency using a genomic DNA (gDNA) dilution series. Primers with the efficiency in the range of 90%–115% were selected as a final panel of primers (Table 1).

To obtain calibrator samples with which all other samples could be compared, we extracted gDNA from two cell lines (H7.s14 and Shef6 8H12) with a known diploid number of chromosomes as assessed by G-banding (Table S2). The calibrator lines were also diploid for 20q11.21 CNV as assessed by FISH (Table S2). We first calculated the copy numbers of loci for calibrator samples (relative to each other) in a qPCR assay and then used three SDs of the copy-number values of the calibrators as a cutoff point for determining the gain or loss of chromosomal regions (red lines on graphs in Figure 4). To validate our assay, we obtained gDNA from a number of cell lines and their subclones (Shef5, MasterShef 8, MasterShef 14, H14.s9, H7.s14-Tomato, H14BJ1, Shef5-SF9, H7.s6, HES3-MIXL, and Shef6 2A7), which were also independently assessed by karyotyping. For each test line Cq values of target genes were first normalized to the reference gene *RELL1* on chromosome 4 for that sample (dCq). The relative quantities of target genes were then calculated relative to the target genes in each of the two calibrator samples (ddCq). The relative amount of target in the sample was calculated as 2^{-ddCq} and the copy numbers were estimated as $2 * 2^{-ddCq}$.

Using such relative quantification, our qPCR analysis detected no abnormalities for tested loci in Shef5 (Figure 4A), MasterShef 8 (Figure 4B), MasterShef 14 (Figure 4C), H14.s9 (Figure 4D), and H7.s14-Tomato (Figure 4E) hPSC lines, as copy-number values for all of the tested target genes were approximately 2. The normal karyotypes of these lines were confirmed by an independent cytogenetic analysis (Table S2). On the other hand, qPCR assay revealed copy-number changes in the H14BJ1 line indicating gains of chromosomes 12, 17, and 20q (Figure 4F). Chromosomes 12, 17q, and 20q were present at three copies, whereas the quantification of copy numbers for chromosome 17p11.2 in the qPCR assay indicated a presence of 33 copies. The huge increase in copy numbers detected by qPCR is consistent with a homogeneous staining region indicating amplification of 17p11.2 seen by G-banding (Figure 4F and Table S2).

Shef5-SF9 line showed gains of chromosome 17p and 20q (Figure 4G). These results were also independently confirmed by karyotyping and FISH for chromosome 20q (Table S2). In the H7.s6 line, we detected a gain of chromosome 1q, 17q, and 20q by qPCR (Figure 4H). The gains of 1q and 17q were consistent with G-banding data showing an abnormal karyotype with an additional structurally abnormal chromosome 1 and an unbalanced

rearrangement between chromosomes 6 and 17, resulting in 17q gain in all cells examined (Table S2). A gain of chromosome 20q was not apparent by G-banding (Table S2).

The qPCR assay for HES3-MIXL line revealed a copy-number change in chromosome 20q (Figure 4I). This result was not apparent by karyotyping, but was confirmed by FISH analysis (Table S2). However, G-banding highlighted an abnormality of chromosome 10 in 2 out of 30 HES3-MIXL cells analyzed, a difference not detected by qPCR as chromosome 10 primers were not included in the panel. Finally, Shef6 2A7 subline also showed a gain of chromosome 20q in the qPCR assay but appeared normal by karyotyping (Figure 4J and Table S2). The validity of the qPCR result was subsequently confirmed by FISH analysis, which revealed 41% of cells with a chromosome 20q gain (Table S2). Thus, for the panel of cells tested qPCR analysis matched the karyotyping and FISH data. A copy-number change in chromosome 20q was detected in four lines, which appeared normal for chromosome 20q by G-banding.

Sensitivity of qPCR Assay versus Digital Droplet PCR and FISH in Detecting Mosaicism in hPSC Cultures

The sensitivity of PCR-based methods is known to depend on the magnitude of the copy-number change, with a difference between zero and one copy being easier to detect than a difference between two and three copies (Whale et al., 2012). We tested this by mixing gDNA of male and female cell lines at different ratios and then performing a qPCR for *SRY* gene on chromosome Y. We observed a significant difference in the copy-number change when male gDNA (with one copy of *SRY*) spiked into the gDNA from female cells (with no copies of *SRY*) was present at as low as 0.5% ($p < 0.05$, Student's t test for sample with 0% male DNA versus the sample with 0.5% male DNA) (Figure S5). However, the majority of aberrations observed in hPSCs (as summarized in Figure 1) resulted in a change in the copy number of chromosomal regions from two copies in the normal diploid cell to three copies in the aneuploid cell. Hence, to determine the sensitivity of the qPCR assay in detecting mosaicism in hPSC cultures, we constructed mosaic samples by mixing diploid cells with increasing numbers of cells that had a complex abnormal karyotype, including a gain of chromosome 17q. After mixing the cells, each sample was split into two tubes, one of which was processed for gDNA extraction and the other for FISH analysis. Copy-number analysis for *TK1* gene (located on chromosome 17q23.2-q25.3) showed an increase in the copy-number values between two copies in the sample with 0% abnormal cells and three copies in 100% abnormal sample (Figure 5A). A significant difference in the copy number compared with 0% control was evident in the samples with 10% abnormal cells ($p < 0.001$; Student's t test for the biological triplicate of copy-number values in 0% versus 10% samples). The same gDNA samples were also tested in the digital droplet PCR (ddPCR) assay on a Bio-Rad QX200 platform, which separates the PCR mixture into 20,000 droplets. The ddPCR was performed using a commercial TaqMan CNV assay for *TK1* and the TaqMan Copy Number Reference Assay for *TERT* (chromosome 5p15.33). A significant difference in the copy-number values in the biological triplicates was noted when abnormal cells were present at 10% or more of the total cell numbers ($p < 0.05$, Student's t test) (Figure 5B). Testing of the same samples on the RainDrop Digital PCR system (RainDance Technologies), which can separate PCR reactions in up to 10 million droplets, allowed detection of the variant cells when they were present at 5% in the mixed samples ($p < 0.01$, Student's t test), but the very low levels of mosaicism were still not detectable (Figure S5B). Finally, we also tested the same set of samples by FISH using a probe at 17q22. The FISH analysis of a 100 interphase cells detected the abnormal cells when they were present at 5% or more ($p < 0.001$, Student's t test for the biological triplicate of 0% versus 5% samples) (Figure 5C). The analysis of 1,000 cells improved the sensitivity of the FISH assay and allowed the abnormal cells to be detected when they were present at 1% ($p < 0.05$, Student's t test for the biological triplicate of 0% versus 1% samples) (Figure 5D).

To determine the sensitivity of the qPCR assay in detecting a smaller structural genetic change, such as the gain of CNV 20q11.21, we mixed clonal sublines with or without the 20q11.21 gain as determined by FISH analysis on interphase cells (data not shown). After mixing the cells each sample was split into two tubes, one of which was processed for gDNA extraction and the other for FISH analysis. Control samples (0% and 100% variant cells) were also tested at the same time. The qPCR assay was able to detect abnormal cells in culture when they were present at around 10%–20% of cells in the mosaic samples across biological triplicates (Figure 6A). The ddPCR assay was performed on the Bio-Rad QX200 platform using commercial TaqMan CNV assay for *BCL2L1* and the *TERT* TaqMan Copy Number Reference Assay. A significant difference in the copy-number values in the biological triplicates was noted when abnormal cells were present at 5% or more of the total cell numbers ($p < 0.005$, Student's t test) (Figure 6B). We also reliably detected abnormal cells by FISH only when they were present at 10% or more in the mixed samples ($p < 0.005$, Student's t test) (Figure 6C). Increasing the number of interphase cells analyzed to 1,000 improved the sensitivity but only down to 5% ($p < 0.05$, Student's t test for 0% versus 5% samples) (Figure 6D). Hence, FISH appeared slightly less sensitive in detecting chromosome 20q CNV than the gain of 17q, possibly due to the difficulty

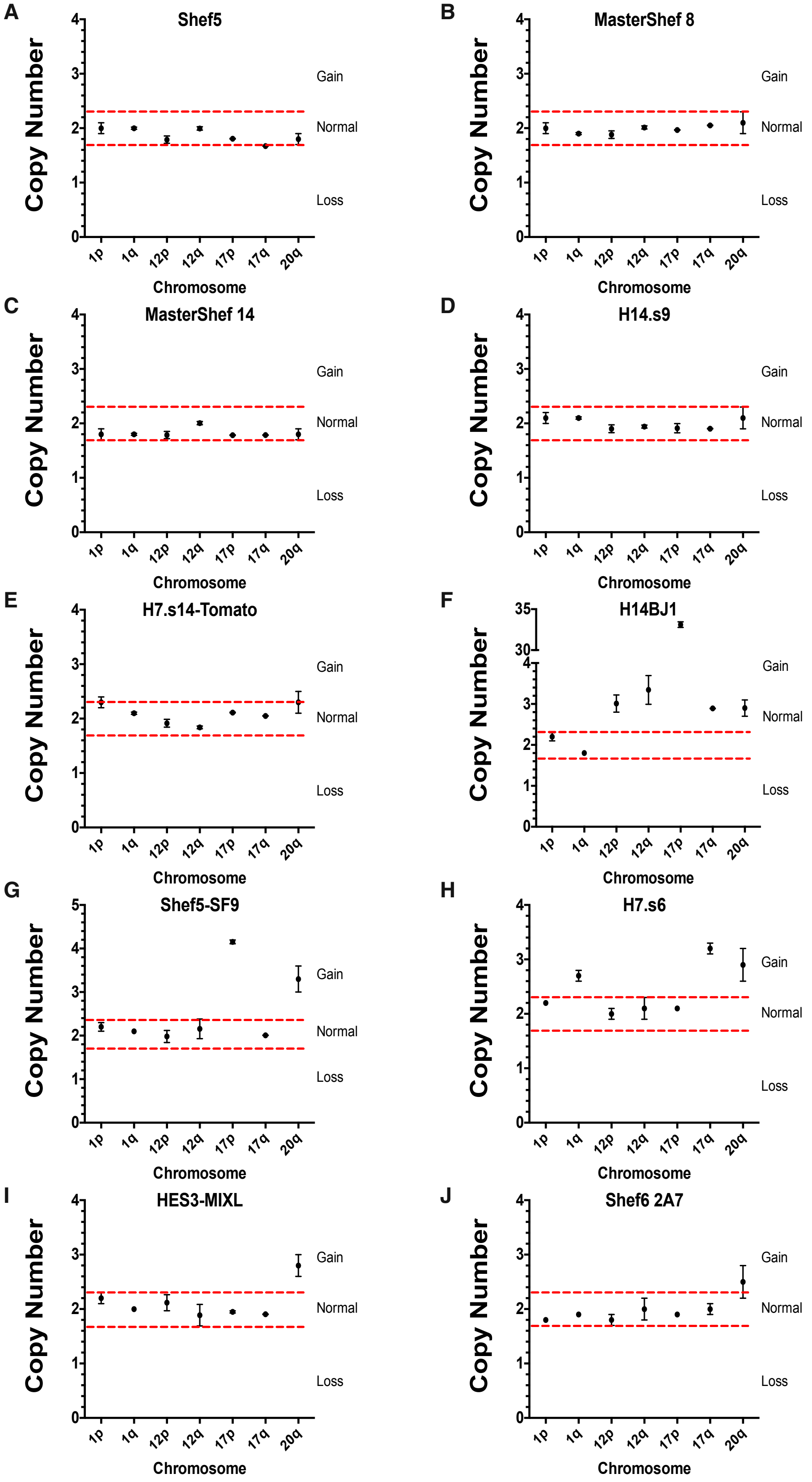

(legend on next page)

in resolving the presence of an additional signal on a tandemly duplicated region.

DISCUSSION

A particular safety concern for the use of hPSCs in regenerative medicine is the presence of genetic changes that occur upon culture. Given the range of commonly occurring changes in hPSCs, it is not possible to recommend a single method that could detect all possible types of changes with an equally high resolution and/or accuracy. Nonetheless, having a good understanding of the caveats associated with particular methods is important for any risk/benefit analysis. Here we investigated the sensitivity of karyotyping, PCR-based methods, and FISH to gain a clear understanding of the advantages and, particularly, limitations of different methods for detecting genetic mosaicism in hPSC cultures.

For karyotyping, we questioned whether the statistical assumptions currently used in clinical cytogenetic practice also apply to hPSCs due to the possible distorting effects of culturing and sampling conditions of hPSCs on the outcome of these tests. Our results from the mixing experiments confirmed that overall the observed numbers of abnormal hPSCs fall within the expected statistical assumptions, and scoring abnormal variants was not substantially distorted by different growth characteristics of the variant cells. In some experiments at higher percentages of abnormal cells in mosaic cultures (>13%), a few of the observed numbers of abnormal cells were higher than the statistically predicted ones. This overestimation of the number of abnormal cells may be due to a difference in cell-cycle time between normal and variant cells. Indeed, we have previously reported a faster cycling time of aneuploid cells compared with their diploid counterparts (Barbaric et al., 2014). As karyotyping relies on cells arrested in metaphase, a different proliferative activity of cells within a mosaic population may bias the analysis toward the more proliferative cells (Gohring et al., 2011). This interpretation is supported by results from FISH on interphase cells from the same samples, where detected numbers of abnormal cells do not fall outside the upper limit of statistically expected numbers (data not shown). Thus, karyotyping of mosaic cultures entailing variant cells with a high proliferative activity would tend to overestimate, rather than underestimate, the presence of variant cells.

Like any other method, karyotyping cannot prove the absence of variant cells. Nonetheless, mosaicism can be excluded to a certain degree by analyzing an appropriate number of metaphases (Hook, 1977). The exact number of metaphases scored may depend on the ultimate application of cells. For example, for routine culturing of hPSCs scoring of 20 or 30 metaphases may be sufficient, as this allows exclusion of 14% or 10% of mosaicism at 95% confidence, respectively. However, detection of very low levels of mosaicism (<1%) would require testing of more than 500 metaphases, which is impractical for routine testing of cultures but may be warranted if the cells are being used in clinical applications. Given this dependence of the sensitivity of karyotyping on the numbers of metaphases analyzed, any karyotyping report and/or published data should clearly indicate the numbers of metaphases scored.

One drawback to karyotyping in the context of routine laboratory practice is the need for expert cytogenetics analyses, which can hamper the frequency of the testing. Yet variant hPSCs with common genetic changes rapidly outcompete normal cells in culture, making early detection and frequent testing for abnormalities essential (Olariu et al., 2010). With this in mind, we designed a qPCR assay for detecting common genetic changes in hPSCs. When tested on a panel of cell lines, our assay accurately reflected the data obtained by karyotyping and/or FISH. The simplicity of the method allows for the data to be obtained within the same day, making it an ideal approach for routine screening of the common changes that occur in hPSC lines. A disadvantage of the qPCR-based assessment (which is also true for FISH) is that the assays can only detect changes at predetermined loci rather than assess the whole genome, and so they do not negate the need for detailed genetic analysis to detect other changes. Nonetheless, due to the non-random changes observed in hPSCs it should be possible to create a panel of primers that would detect the majority of the changes. We estimated that the panel of primers used in this study would detect about 45% of the reported genetic changes summarized in Figure 1. The flexibility of the method allows for further primer pairs to be designed to cover additional regions in the genome, although this would increase the overall cost of screening.

Figure 4. qPCR Assay for Detecting Common Genetic Changes in hPSCs
Copy-number values for target genes on commonly amplified chromosomal regions for the hPSC lines (A) Shef5, (B) MasterShef 8, (C) MasterShef 14, (D) H14.s9, (E) H7.s14-Tomato, (F) H14BJ1, (G) Shef5-SF9, (H) H7.s6, (I) HES3-MIXL, and (J) Shef6 2A7. Plotted values are means of copy numbers calculated relative to each of the calibrator lines ± SEM. Red lines represent cutoff levels calculated as 3 SDs of the copy-number values of calibrator samples.

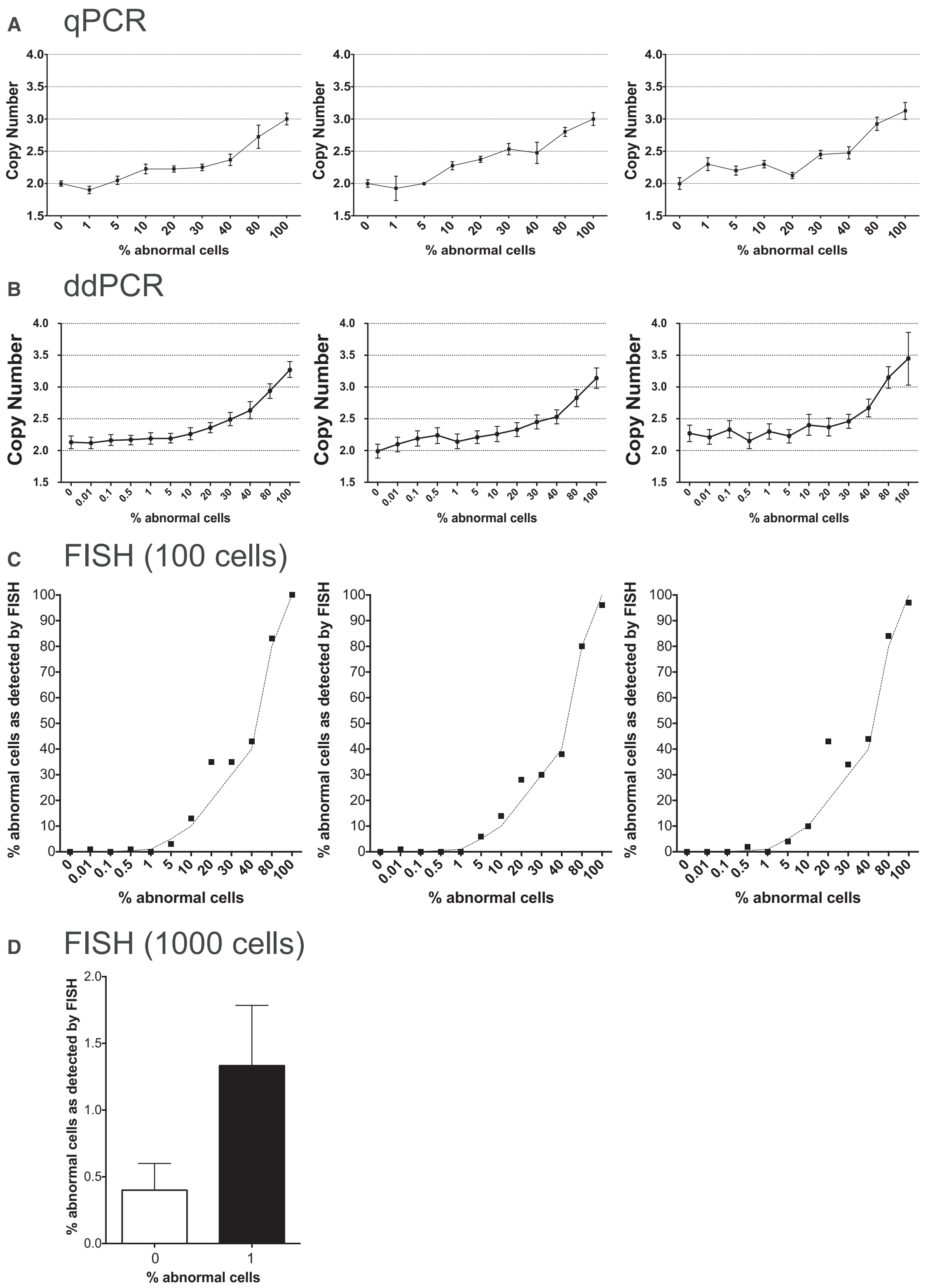

Figure 5. Sensitivity of Different Methods for Detecting Mosaicism of Chromosome 17q Trisomy in hPSC Cultures

(A) qPCR analysis of samples with ratios of abnormal cells ranging from 0% to 100%. The panels from left to right represent three biological replicates. Plotted values on each graph are copy-number means of technical triplicates ± SEM.

(legend continued on next page)

Unlike G-banding, which generally may not be able to detect changes smaller than 5 Mb, qPCR and FISH are useful for detecting small amplifications and deletions. A case in point is the gain of chromosome 20q11.21. The gain of 20q11.21 is a particularly insidious genetic aberration as it occurs in many cell lines with no overt karyotypic changes (Amps et al., 2011). The 20q11.21 gain appears relatively frequently and, once acquired, confers selective advantage to variant cells, which rapidly overtake the culture (Amps et al., 2011; Avery et al., 2013). Thus, hPSC cultures should be regularly tested for the chromosome 20q CNV. Whereas FISH and karyotyping typically require specialist staff and often must be outsourced at costs of up to US$850, the qPCR can usually be performed in-house for the cost of reagents.

The sensitivity of the qPCR assay of around 10% for copy-number changes allows genetic variants to be detected before they overtake the cultures and mosaic cultures can be either discarded or recloned. We did not achieve an improvement in the sensitivity below 5% when using ddPCR. The sensitivity of the PCR-based methods in detecting copy-number changes depends on both the magnitude of the copy-number change and the frequency of the abnormal cells in mosaic samples. Indeed, the detection of a new haplotype in the mosaic sample allows detection of a copy-number change by qPCR even when variant cells are present in as low as 0.5% of the sample, as we demonstrated using a dilution series of a sample with one copy of chromosome Y. However, majority of genetic changes in hPSCs entail a change in the copy numbers from two to three copies, which would equate to a 50% discrimination between pure populations of triploid cells compared with the diploid counterparts (1.5:1). However, when the ratio of variant cells decreases to 10% of the total cells in mosaic cultures, the assay would require discrimination of only 5% (1.05:1), and at 1% mosaicism of only 0.5% (1.005:1). Although we were unable to achieve the sensitivity of less than 5% with standard protocols used in ddPCR, further optimization (e.g., using increasing amounts of DNA or increased number of assays in commonly gained regions) may allow for improved sensitivity.

The sensitivity of 5%–10% obtained by PCR-based methods and FISH may be suitable for routine culturing of cells, but the applications of hPSCs in the clinic will likely require a more rigorous sensitivity. Some of the alternative methods for detection of genetic aberrations in hPSCs that were not included in our study include comparative genomic hybridization (CGH) or SNP array platforms, e-karyotyping, and next-generation sequencing. Previous studies on the sensitivity of CGH and SNP arrays in detecting mosaicism in various clinical samples estimated the sensitivity of 8%–20% (Ballif et al., 2006; Cross et al., 2007; Valli et al., 2011), whereas an estimated sensitivity of e-karyotyping is around 30% (Ben-David et al., 2013).

Comprehensive genomic profiling is likely to uncover a range of genetic aberrations in a preparation of cells. However, of those changes that are acquired during culture of hPSCs many appear to confer selective growth advantage to the undifferentiated cells, but their consequences for behavior of particular differentiated cells remain unknown. Overall, our evaluation of the commonly employed methods for genetic testing of hPSCs revealed their limit of mosaicism detection to be around 5%–10%. Thus, a preparation of cells could be declared "genetically normal" but nevertheless harbor small populations of variant cells, the consequences of which for an experimental outcome or transplantation to a patient remain unknown. Recognition of this point is crucial for developing strategies for routine laboratory practice as well as the regulation for the use of hPSCs in regenerative medicine. New strategies for detecting mosaicism may help, although it is unlikely that even whole-genome systems can be made sufficiently sensitive to eliminate the possibility of mosaicism. Thus, it becomes essential to assess the consequences of specific changes, particularly those that occur commonly, for the functional behavior of particular differentiated cells. Assessment of potential risks will then depend on cell types in question and the types of application.

EXPERIMENTAL PROCEDURES

Human Pluripotent Stem Cell Culture

Stock cultures of hPSC lines (see Supplemental Experimental Procedures) were maintained at 37°C under a humidified atmosphere of 5% CO_2 in air. For routine maintenance, cells were grown either on Matrigel (BD Biosciences) in mTESR medium (StemCell Technologies), or on vitronectin (Life Technologies) in Essential 8 (Life Technologies), or on CELLStart (Life Technologies) in NutriStem medium (Stemgent).

(B) ddPCR analysis of samples with ratios of abnormal cells ranging from 0% to 100%. The panels represent three biological replicates. Plotted on each graph are copy-number values and the Poisson distribution at 95% confidence interval.
(C) FISH analysis of samples containing 0%–100% cells with a gain of chromosome 17q. The panels represent three biological replicates. Plotted values on each graph are percentage of cells with amplification among 100 cells analyzed. Dotted lines on each graph represent the expected values for each mixed sample.
(D) FISH analysis of samples containing 0% and 1% cells with a gain of chromosome 17q, shown as the percentage of cells with amplification among 1,000 cells analyzed. Plotted values are averages of biological triplicates ± SD.

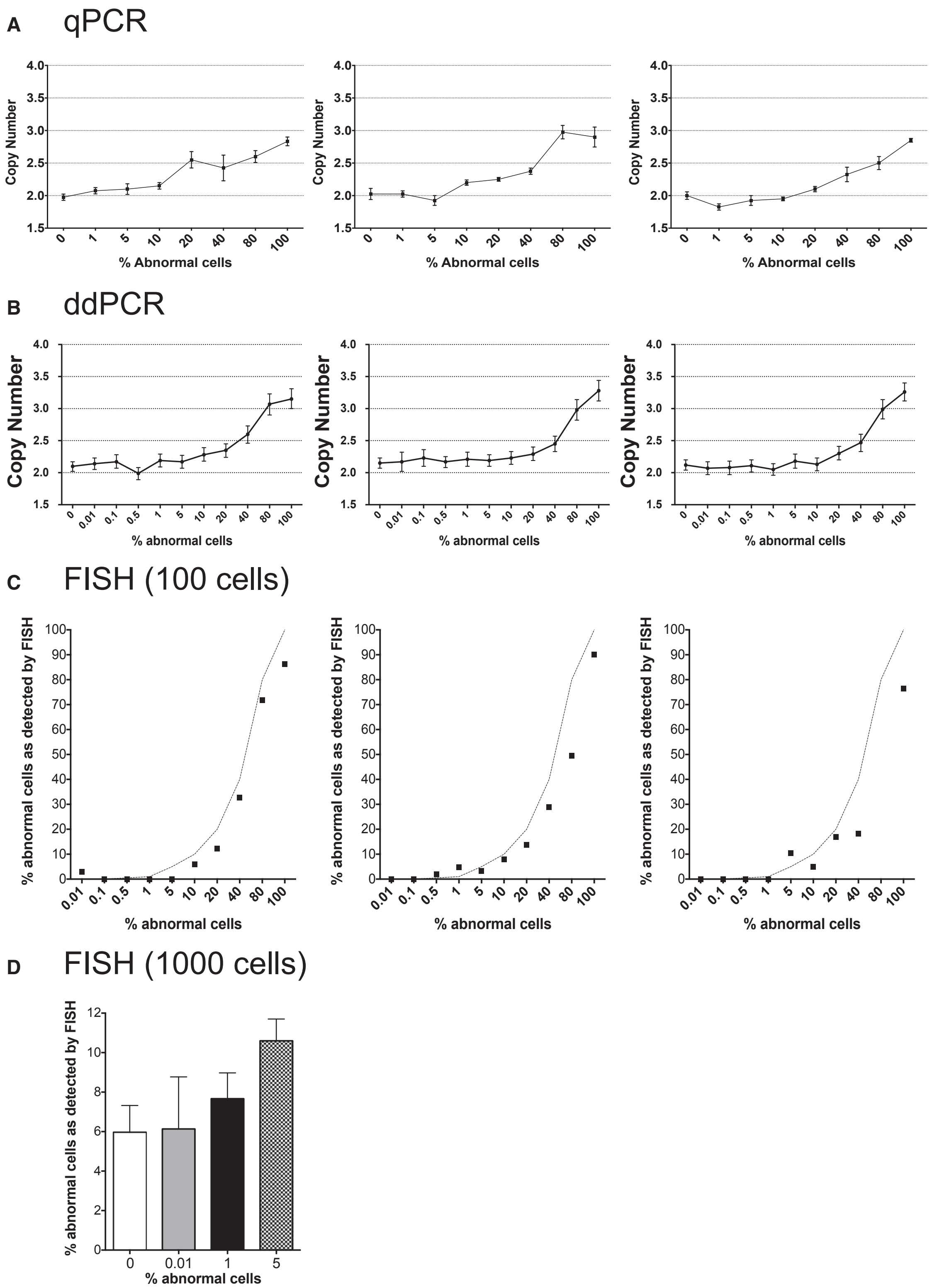

Figure 6. Sensitivity of Different Methods for Detecting Mosaicism of Chromosome 20q11.21 CNV in hPSC Cultures

(A) qPCR analysis of samples with ratios of abnormal cells ranging from 0% to 100%. The panels represent three biological replicates. Plotted values on each graph are copy-number means of technical triplicates ± SEM.

(legend continued on next page)

Fluorescence In Situ Hybridization

The cells were pelleted via centrifugation and resuspended in prewarmed 0.0375 M KCl solution and incubated for 10 min. Following centrifugation the cells were resuspended in fixative (3:1 methanol/acetic acid). One drop of suspension was dropped onto a glass microscope slide (Sigma). The *BCL2L1* FISH probe was a spectrum green fluorescently labeled BAC (RP5-857M17, almost 100 kb) provided by BlueGnome (Illumina), and covers the genes *BCL2L1*, *COX4I2*, and the 3′ end of *ID1*. For 20q-telomere detection, the Vysis (Abbott Molecular) probes TelVysion 20q-telomere (spectrum orange) were used. For detection of chromosome 17p/q copy number, the Kreatech ISO 17q (specific for the *TP53* gene at 17p13 labeled red and the *MPO* gene at 17q22 labeled green) probes were used. Probe and slides were denatured together at 72°C for 2 min in a PTC-200 DNA Engine (Peltier Thermal Cycler, MJ Research) and incubated at 37°C for 16 hr for hybridization. Slides were washed in 0.4× sodium citrate (Abbott Molecular) with 0.3% Tween 20 (Sigma) and 2× sodium citrate with 0.1% Tween 20. Coverslips were mounted on the slides in Vectashield Mounting Medium with DAPI (Vector Laboratories). Either 100 or 1,000 interphase cells were analyzed on an Olympus BX51 fluorescent microscope, as indicated.

Mixing Experiments for Detecting the Sensitivity of Karyotyping

Diploid hPSC sublines (H7.s14, H7.s14-Tomato, or H14.s9) were mixed at different ratios with their aneuploid counterparts (H7.s6-GFP or H14BJ1-GFP) as detailed in Supplemental Experimental Procedures. Ratios of abnormal cells in culture were assessed either by high-content imaging using the InCell Analyzer 1000 (GE Healthcare) and associated image analysis software (Developer Toolbox 1.7, GE Healthcare) or by flow cytometry of a duplicate flask using BD FACSJazz (BD Biosciences). Mosaic cultures were treated with colcemid and processed for G-banding (Supplemental Experimental Procedures).

Mixing Experiments for Detecting Sensitivity of PCR-Based Methods and FISH

For testing the sensitivity of methods in detecting a gain of chromosome 17q, we used H7.s14 diploid subline and H7.s6-GFP aneuploid subline trisomic for chromosome 17q. For chromosome 20q sensitivity, we used clonal Shef6 8H12 and Shef6 2A subline, diploid and trisomic for 20q, respectively. All sublines were grown on Matrigel in mTESR prior to the experiment and then dissociated using trypsin and counted. After mixing the cells to achieve 0.01%–100% of abnormal cells within the diploid population, each mosaic sample was split into two tubes, one of which was processed for FISH analysis and the other one for gDNA extraction. The control samples (0% variant cells) were also tested at the same time. For testing the sensitivity of qPCR assay in detecting a change of copy numbers from 0 to 1, we spiked gDNA from a male hPSC line (MasterShef 8, 46,XY) into gDNA from a female line (MasterShef 14, 46,XX) in a range of ratios from 0.01% to 100%. qPCR was used to detect a copy number of *SRY* (chromosome Yp11.32). The sequences of the forward and reverse primers used in the qPCR reaction were aaaattggcgattaagtcaaattc and ctgcctccctgactgctct, respectively. The qPCR was performed in 10-μL reactions as detailed below.

qPCR for Determining Copy-Number Changes of Target Genes

Primers for qPCR were designed and validated as detailed in Supplemental Experimental Procedures. qPCR was performed in 10-μL reactions in triplicates or quadruplicates, using gene-specific primers (Table 1), probes from the Universal Probe Library (Table 1), and TaqMan Fast Universal PCR Master Mix (Life Technologies). Each PCR reaction contained 1× manufacturer's buffer, 100 nM of each of the forward and reverse primers, and 10 or 20 ng of DNA template. No template controls were also included on each plate. Samples were heated to 50°C for 2 min and denatured at 95°C for 10 min. This was followed by 40 cycles of 95°C for 15 s and 60°C for 1 min. Reactions were run on 384-well plates on the QuantStudio 12K Flex Real-Time PCR System (Applied Biosystems, Life Technologies). The Cq values were obtained from the QuantStudio 12K Flex Software with auto baseline settings and were then exported to Excel for copy-number analysis using the relative quantification method (2^{-ddCq}) (as detailed in Supplemental Experimental Procedures).

Digital Droplet PCR on the Bio-Rad QX200 Platform

A copy-number variation experiment was performed using a TaqMan assay specific to *BCL2L1* (Life Technologies) or *TK1* (Life Technologies) against the VIC-labeled reference assay (*TERT*) (Life Technologies) set at two copies (CNV2) on the Bio-Rad QX200 ddPCR system (Bio-Rad). Reaction mixtures (20 μL) contained 1 μL of DNA extract, 1× ddPCR Supermix for probes (Bio-Rad), 225 nM of each primer, and 50 nM of each probe. Reaction mixes were loaded either into DG8 cartridges together with 70 μL of droplet oil per sample; droplets were generated using the QX100 Droplet Generator or loaded in plate format into the Bio-Rad QX200 AutoDG and generated as per the manufacturer's instructions. The oil/reagent emulsion was transferred to a 96-well semi-skirted plate (Eppendorf) and the samples were amplified on the Bio-Rad C1000 Touch thermocycler (95°C for 10 min, followed by 40 cycles of 94°C for 30 s and 60°C for 60 s, with a final elongation step of 98°C for 10 min). The plate containing the

(B) ddPCR analysis of samples with ratios of abnormal cells ranging from 0% to 100%. The panels represent three biological replicates. Plotted on each graph are copy-number values and the Poisson distribution at 95% confidence interval.
(C) FISH analysis of samples containing 0%–100% cells with a gain of chromosome 20q. The panels represent three biological replicates. Plotted values on each graph are percentage of cells with amplification among 100 cells analyzed. Dotted lines on each graph represent the expected values for each mixed sample.
(D) FISH analysis of samples containing 0%, 0.01%, 1%, and 5% of cells with a gain of chromosome 20q, shown as the percentage of cells with amplification among 1,000 cells analyzed. Plotted values are averages of biological triplicates ± SD.

droplet amplicons was subsequently loaded into the QX200 Droplet Reader (Bio-Rad). Analysis was performed using Quantasoft software (Bio-Rad).

Statistical Analysis

Data were statistically analyzed using Excel. $p < 0.05$ defined statistical significance. To test the statistical difference between different levels of mosaicism across biological replicates in the ddPCR, we first normalized 0% samples within each biological replicate to 2 and normalized the mosaic samples to the 0% sample.

SUPPLEMENTAL INFORMATION

Supplemental Information includes Supplemental Experimental Procedures, five figures, and two tables and can be found with this article online at http://dx.doi.org/10.1016/j.stemcr.2016.10.003.

AUTHOR CONTRIBUTIONS

D.B., A.J.H., M.J., P.W.A., and I.B. conceived and designed experiments. D.B., A.J.H., P.J.G., M.J., S.W., M.W., K.B., T.F.A., and I.B. performed the experiments and/or analyzed the data. H.D.M. derived some of the hPSC lines used in the study. D.B., A.J.H., P.J.G., M.J., P.W.A., and I.B. wrote the manuscript.

ACKNOWLEDGMENTS

We would like to thank MRC Harwell for technical services, in particular Dr. Gemma Codner and Dr. Sara Wells. This work was supported by the UK Regenerative Medicine Platform (grant number MR/L012537/1) and the Medical Research Council (grant number MR/N009371/1).

Received: March 29, 2016
Revised: October 10, 2016
Accepted: October 10, 2016
Published: November 8, 2016

REFERENCES

Amps, K., Andrews, P.W., Anyfantis, G., Armstrong, L., Avery, S., Baharvand, H., Baker, J., Baker, D., Munoz, M.B., Beil, S., et al. (2011). Screening ethnically diverse human embryonic stem cells identifies a chromosome 20 minimal amplicon conferring growth advantage. Nat. Biotechnol. *29*, 1132–1144.

Avery, S., Hirst, A.J., Baker, D., Lim, C.Y., Alagaratnam, S., Skotheim, R.I., Lothe, R.A., Pera, M.F., Colman, A., Robson, P., et al. (2013). BCL-XL mediates the strong selective advantage of a 20q11.21 amplification commonly found in human embryonic stem cell cultures. Stem Cell Rep. *1*, 379–386.

Baker, D.E., Harrison, N.J., Maltby, E., Smith, K., Moore, H.D., Shaw, P.J., Heath, P.R., Holden, H., and Andrews, P.W. (2007). Adaptation to culture of human embryonic stem cells and oncogenesis in vivo. Nat. Biotechnol. *25*, 207–215.

Ballif, B.C., Rorem, E.A., Sundin, K., Lincicum, M., Gaskin, S., Coppinger, J., Kashork, C.D., Shaffer, L.G., and Bejjani, B.A. (2006). Detection of low-level mosaicism by array CGH in routine diagnostic specimens. Am. J. Med. Genet. A. *140*, 2757–2767.

Barbaric, I., Biga, V., Gokhale, P.J., Jones, M., Stavish, D., Glen, A., Coca, D., and Andrews, P.W. (2014). Time-lapse analysis of human embryonic stem cells reveals multiple bottlenecks restricting colony formation and their relief upon culture adaptation. Stem Cell Rep. *3*, 142–155.

Ben-David, U., Mayshar, Y., and Benvenisty, N. (2013). Virtual karyotyping of pluripotent stem cells on the basis of their global gene expression profiles. Nat. Protoc. *8*, 989–997.

Blum, B., Bar-Nur, O., Golan-Lev, T., and Benvenisty, N. (2009). The anti-apoptotic gene survivin contributes to teratoma formation by human embryonic stem cells. Nat. Biotechnol. *27*, 281–287.

Cross, J., Peters, G., Wu, Z., Brohede, J., and Hannan, G.N. (2007). Resolution of trisomic mosaicism in prenatal diagnosis: estimated performance of a 50K SNP microarray. Prenat. Diagn. *27*, 1197–1204.

D'Hulst, C., Parvanova, I., Tomoiaga, D., Sapar, M.L., and Feinstein, P. (2013). Fast quantitative real-time PCR-based screening for common chromosomal aneuploidies in mouse embryonic stem cells. Stem Cell Rep. *1*, 350–359.

Draper, J.S., Smith, K., Gokhale, P., Moore, H.D., Maltby, E., Johnson, J., Meisner, L., Zwaka, T.P., Thomson, J.A., and Andrews, P.W. (2004). Recurrent gain of chromosomes 17q and 12 in cultured human embryonic stem cells. Nat. Biotechnol. *22*, 53–54.

Enver, T., Soneji, S., Joshi, C., Brown, J., Iborra, F., Orntoft, T., Thykjaer, T., Maltby, E., Smith, K., Abu Dawud, R., et al. (2005). Cellular differentiation hierarchies in normal and culture-adapted human embryonic stem cells. Hum. Mol. Genet. *14*, 3129–3140.

Fazeli, A., Liew, C.G., Matin, M.M., Elliott, S., Jeanmeure, L.F., Wright, P.C., Moore, H., and Andrews, P.W. (2011). Altered patterns of differentiation in karyotypically abnormal human embryonic stem cells. Int. J. Dev. Biol. *55*, 175–180.

Gekas, J., van den Berg, D.G., Durand, A., Vallee, M., Wildschut, H.I., Bujold, E., Forest, J.C., Rousseau, F., and Reinharz, D. (2011). Rapid testing versus karyotyping in Down's syndrome screening: cost-effectiveness and detection of clinically significant chromosome abnormalities. Eur. J. Hum. Genet. *19*, 3–9.

Gohring, G., Giagounidis, A., Busche, G., Hofmann, W., Kreipe, H.H., Fenaux, P., Hellstrom-Lindberg, E., and Schlegelberger, B. (2011). Cytogenetic follow-up by karyotyping and fluorescence in situ hybridization: implications for monitoring patients with myelodysplastic syndrome and deletion 5q treated with lenalidomide. Haematologica *96*, 319–322.

Goldring, C.E., Duffy, P.A., Benvenisty, N., Andrews, P.W., Ben-David, U., Eakins, R., French, N., Hanley, N.A., Kelly, L., Kitteringham, N.R., et al. (2011). Assessing the safety of stem cell therapeutics. Cell Stem Cell *8*, 618–628.

Harrison, N.J., Baker, D., and Andrews, P.W. (2007). Culture adaptation of embryonic stem cells echoes germ cell malignancy. Int. J. Androl. *30*, 275–281, discussion 281.

Hoebeeck, J., Speleman, F., and Vandesompele, J. (2007). Real-time quantitative PCR as an alternative to Southern blot or fluorescence in situ hybridization for detection of gene copy number changes. Methods Mol. Biol. *353*, 205–226.

Hook, E.B. (1977). Exclusion of chromosomal mosaicism: tables of 90%, 95% and 99% confidence limits and comments on use. Am. J. Hum. Genet. *29*, 94–97.

Lefort, N., Feyeux, M., Bas, C., Feraud, O., Bennaceur-Griscelli, A., Tachdjian, G., Peschanski, M., and Perrier, A.L. (2008). Human embryonic stem cells reveal recurrent genomic instability at 20q11.21. Nat. Biotechnol. *26*, 1364–1366.

Martins-Taylor, K., Nisler, B.S., Taapken, S.M., Compton, T., Crandall, L., Montgomery, K.D., Lalande, M., and Xu, R.H. (2011). Recurrent copy number variations in human induced pluripotent stem cells. Nat. Biotechnol. *29*, 488–491.

Olariu, V., Harrison, N.J., Coca, D., Gokhale, P.J., Baker, D., Billings, S., Kadirkamanathan, V., and Andrews, P.W. (2010). Modeling the evolution of culture-adapted human embryonic stem cells. Stem Cell Res. *4*, 50–56.

Olde Nordkamp, L., Mellink, C., van der Schoot, E., and van den Berg, H. (2009). Karyotyping, FISH, and PCR in acute lymphoblastic leukemia: competing or complementary diagnostics? J. Pediatr. Hematol. Oncol. *31*, 930–935.

Sandberg, A.A., Meloni, A.M., and Suijkerbuijk, R.F. (1996). Reviews of chromosome studies in urological tumors. III. Cytogenetics and genes in testicular tumors. J. Urol. *155*, 1531–1556.

L.G. Shaffer, J. McGowan-Jordan, and M. Schmid, eds. (2013). ISCN 2103: An International System for Human Cytogenetic Nomenclature (S. Karger).

Taapken, S.M., Nisler, B.S., Newton, M.A., Sampsell-Barron, T.L., Leonhard, K.A., McIntire, E.M., and Montgomery, K.D. (2011). Karotypic abnormalities in human induced pluripotent stem cells and embryonic stem cells. Nat. Biotechnol. *29*, 313–314.

Takahashi, K., Tanabe, K., Ohnuki, M., Narita, M., Ichisaka, T., Tomoda, K., and Yamanaka, S. (2007). Induction of pluripotent stem cells from adult human fibroblasts by defined factors. Cell *131*, 861–872.

Thomson, J.A., Itskovitz-Eldor, J., Shapiro, S.S., Waknitz, M.A., Swiergiel, J.J., Marshall, V.S., and Jones, J.M. (1998). Embryonic stem cell lines derived from human blastocysts. Science *282*, 1145–1147.

Valli, R., Marletta, C., Pressato, B., Montalbano, G., Lo Curto, F., Pasquali, F., and Maserati, E. (2011). Comparative genomic hybridization on microarray (a-CGH) in constitutional and acquired mosaicism may detect as low as 8% abnormal cells. Mol. Cytogenet. *4*, 13.

Vogelstein, B., and Kinzler, K.W. (1999). Digital PCR. Proc. Natl. Acad. Sci. USA *96*, 9236–9241.

Werbowetski-Ogilvie, T.E., Bosse, M., Stewart, M., Schnerch, A., Ramos-Mejia, V., Rouleau, A., Wynder, T., Smith, M.J., Dingwall, S., Carter, T., et al. (2009). Characterization of human embryonic stem cells with features of neoplastic progression. Nat. Biotechnol. *27*, 91–97.

Whale, A.S., Huggett, J.F., Cowen, S., Speirs, V., Shaw, J., Ellison, S., Foy, C.A., and Scott, D.J. (2012). Comparison of microfluidic digital PCR and conventional quantitative PCR for measuring copy number variation. Nucleic Acids Res. *40*, e82.

Yang, S., Lin, G., Tan, Y.Q., Zhou, D., Deng, L.Y., Cheng, D.H., Luo, S.W., Liu, T.C., Zhou, X.Y., Sun, Z., et al. (2008). Tumor progression of culture-adapted human embryonic stem cells during long-term culture. Genes Chromosomes Cancer *47*, 665–679.

Stem Cell Reports, Volume 7

Supplemental Information

Detecting Genetic Mosaicism in Cultures of Human Pluripotent Stem Cells

Duncan Baker, Adam J. Hirst, Paul J. Gokhale, Miguel A. Juarez, Steve Williams, Mark Wheeler, Kerry Bean, Thomas F. Allison, Harry D. Moore, Peter W. Andrews, and Ivana Barbaric

Supplementary Figure S1.

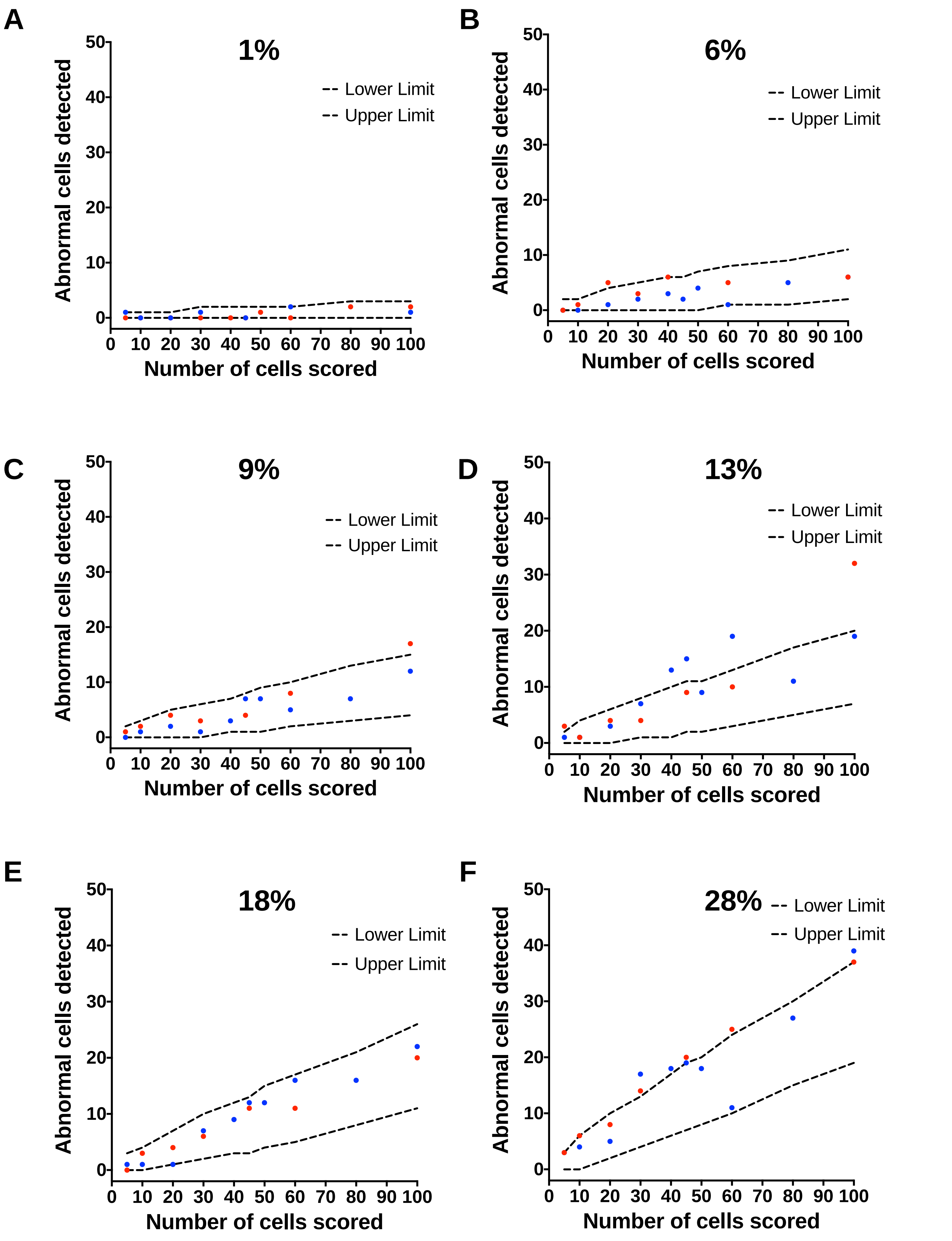

Supplementary Figure S2.

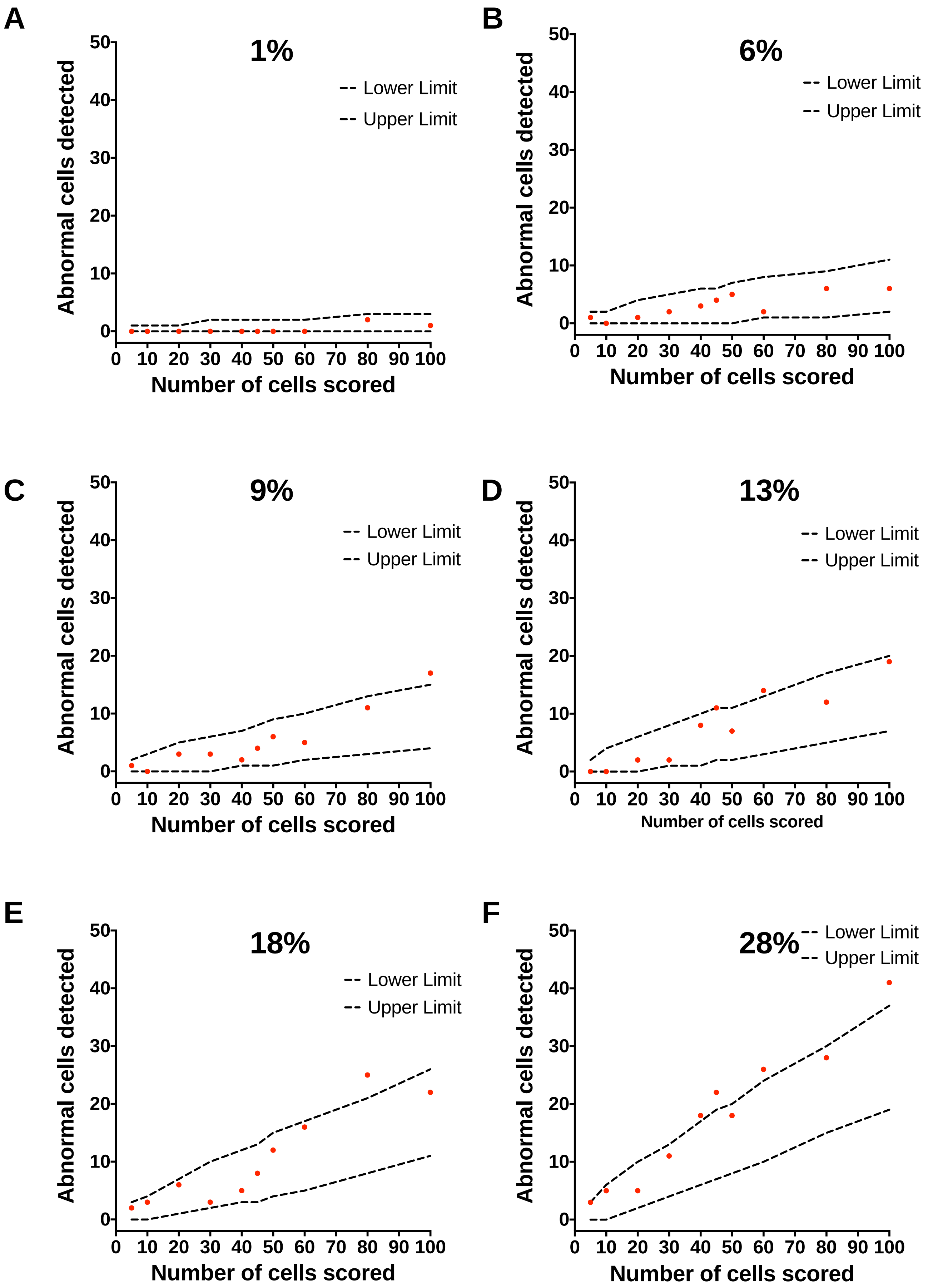

Supplementary Figure S3.

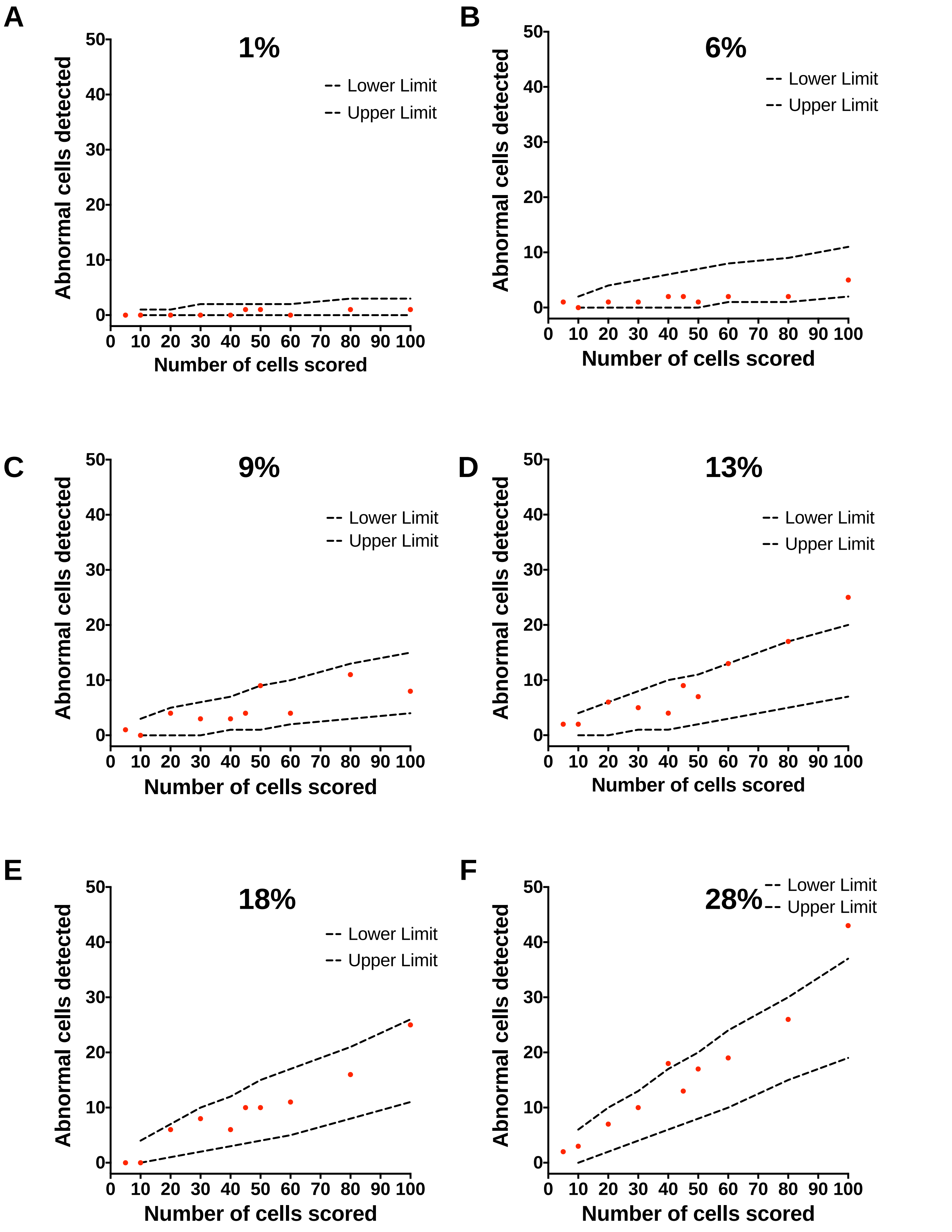

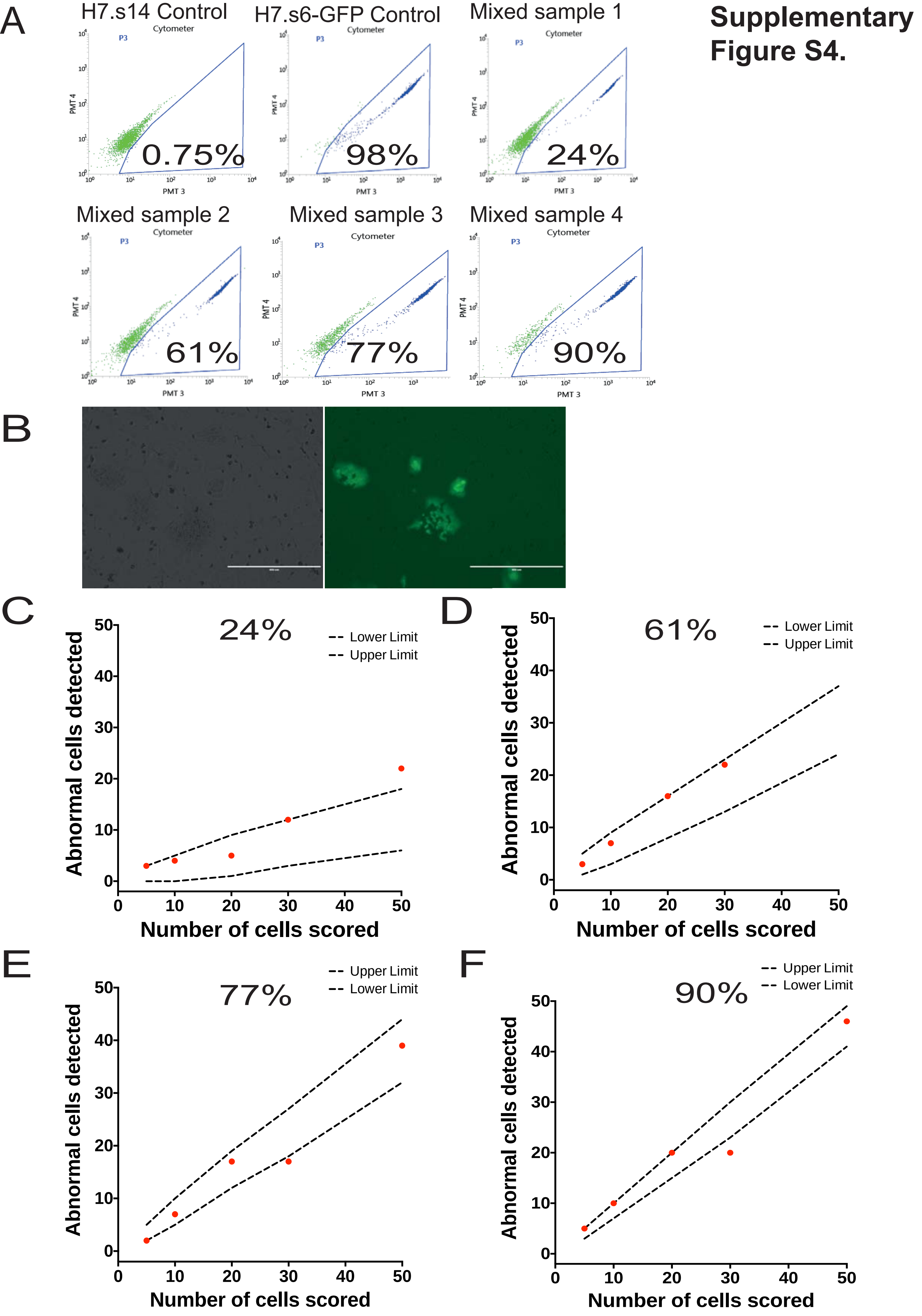

Supplementary Figure S4.
A
H7.s14 Control
H7.s6-GFP Control
Mixed sample 1
Mixed sample 2
Mixed sample 3
Mixed sample 4
Cytometer
P3
PMT 4
PMT 3
0.75%
98%
24%
61%
77%
90%
B
C
D
E
F
Abnormal cells detected
Number of cells scored
Lower Limit
Upper Limit

Supplementary Figure S5.

A

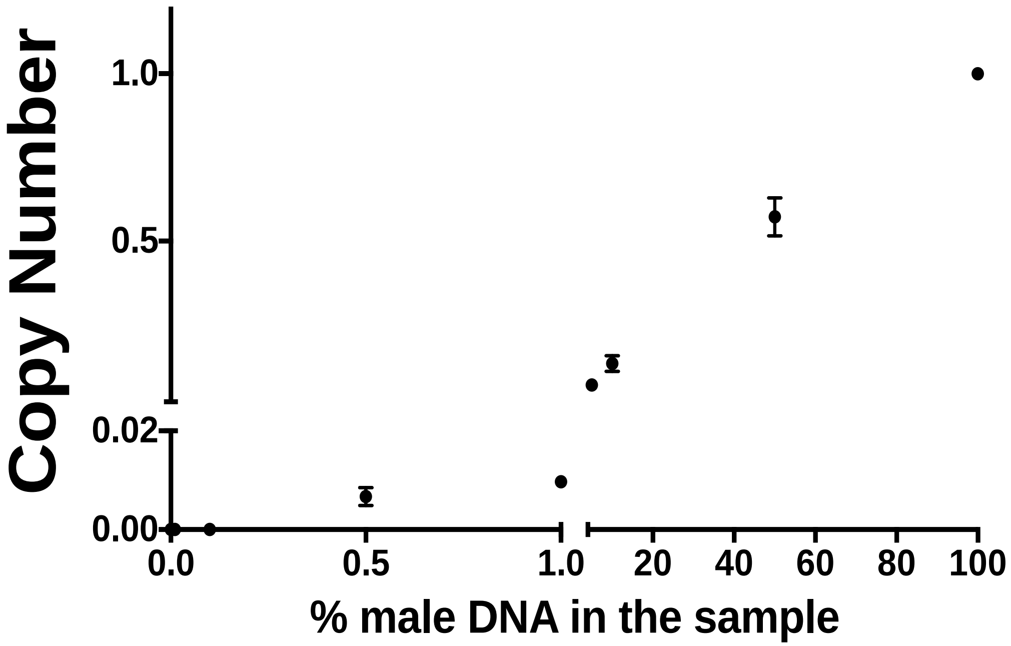

B

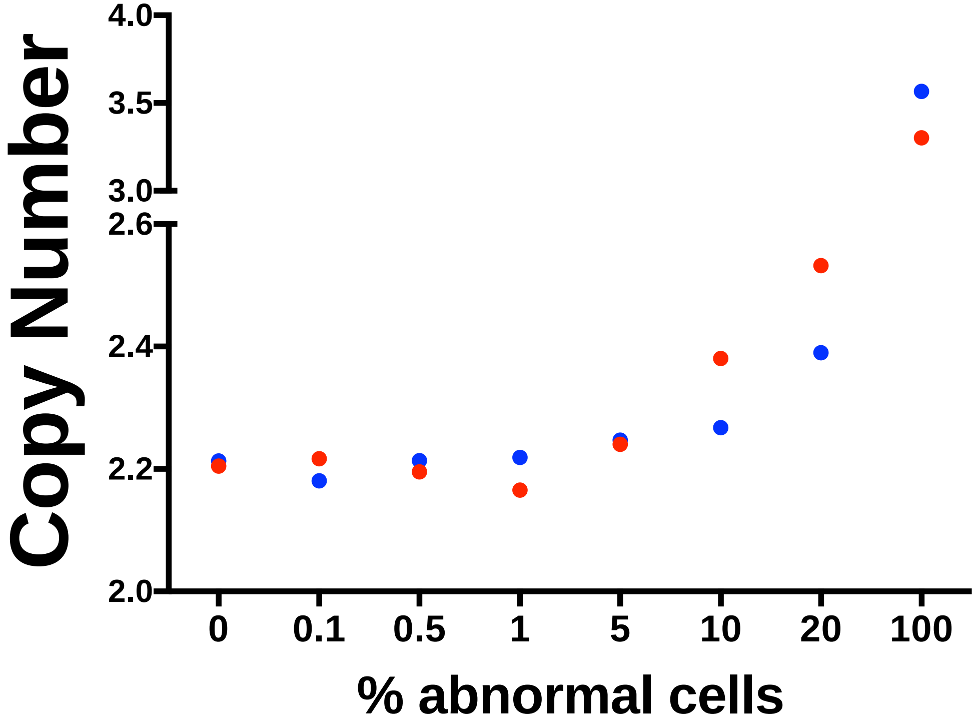

SUPPLEMENTAL INFORMATION

Supplemental Figure Legends

Figure S1. The sensitivity of karyotyping in detecting mosaicism in hPSC cultures: analysis of the samples by a second clinical cytogeneticist; related to Figure 2. Assessment of karyotyping sensitivity was performed using the same prepared slides of mosaic samples as in Figure 1 but the analysis was performed by a different cytogeneticist. Mixed samples containing (A) 1%, (B) 6%, (C) 9%, (D) 13%, (E) 18% and (F) 28% aneuploid cells (H7.s6-GFP) within the diploid cell population (H7.s14) were scored for the presence or absence of the abnormal chromosome 6, der(6)t(6;17)(q27;q1). Increasing numbers of metaphases (from five to 100) were scored from each sample. The numbers of abnormal cells detected in the duplicate analysis (red and blue circles) were plotted against statistically-determined expected numbers of abnormal cells (dotted lines) as detailed in the Supplementary Table S1.

Figure S2. The sensitivity of karyotyping in detecting mosaicism in hPSC cultures: analysis of new slides; related to Figure 2. New slides were prepared from the same mixed samples as analysed in Figures 1 and S1. Mixed samples containing (A) 1%, (B) 6%, (C) 9%, (D) 13%, (E) 18% and (F) 28% aneuploid cells (H7.s6-GFP) within the diploid cell population (H7.s14) were scored for the presence or absence of the abnormal chromosome 6, der(6)t(6;17)(q27;q1). Increasing numbers of metaphases (from five to 100) were scored from each sample. The numbers of abnormal cells detected (red circles) were plotted against statistically-determined expected numbers of abnormal cells (dotted lines) as detailed in the Supplementary Table S1.

Figure S3. The sensitivity of karyotyping in detecting mosaicism in hPSC cultures: a repeat of the experiment using fluorescently labelled H7.s14-Tomato subline; related to Figure 2. Diploid cells (H7.s14-Tomato, stably expressing tdTomato) and their aneuploid counterparts (H7.s6-GFP, stably expressing GFP) were mixed at different ratios (1-28%). The percentage of aneuploid cells was confirmed by imaging the cells on a high-content microscopy platform and using an associated image analysis software to count the number of GFP-expressing cells (H7.s6-GFP) within the total number of cells in the culture (total number of tdTomato-expressing and GFP-expressing cells). Mixed samples containing (A) 1%, (B) 6%, (C) 9%, (D) 13%, (E) 18% and (F) 28% aneuploid cells (H7.s6-GFP) within the diploid cell population (H7.s14-Tomato) were scored for the presence or absence of the abnormal chromosome 6, der(6)t(6;17)(q27;q1). Increasing numbers of metaphases (from five to 100) were scored from each sample. The numbers of abnormal cells detected in the duplicate analysis (red and blue circles) were plotted against statistically-determined expected numbers of abnormal cells (dotted lines) as detailed in the Supplementary Table S1.

Figure S4. The sensitivity of karyotyping in detecting mosaicism in hPSC cultures: a repeat of the experiment using a different way of mixing and counting cells; related to Figures 2 and 3. Diploid (H7.s14) and aneuploid (H7.s6-GFP, stably expressing GFP) cells were dissociated into single cells and mixed at different ratios. Mixed cultures were passaged once into two parallel flasks. (A) Flow cytometry was used to determine the ratio of abnormal cells in mixed cultures based on their GFP expression. The H7.s14 cells only (left panel) were used to set the gate and H7.s6-GFP cells only were used as a control. (B) Representative images of mixed cultures of H7.s14 diploid and H7.s6-GFP aneuploid cells containing 24% abnormal cells. Scale bar represents 400µm. Mixed samples containing (C) 24%, (D) 61%, (E) 77% and (F) 90% aneuploid cells (H7.s6-GFP) within the diploid cell population (H7.s14)

were scored for the presence or absence of the abnormal chromosome 6, der(6)t(6;17)(q27;q1). Increasing numbers of metaphases (from five to 50) were scored from each sample. The numbers of abnormal cells detected (red circles) were plotted against statistically-determined expected numbers of abnormal cells (dotted lines) as detailed in the Supplementary Table S1.

Figure S5. The sensitivity of the PCR-based assays in detecting mosaicism; related to Figures 5 and 6. (A) The sensitivity of qPCR for detecting copy number changes from zero to one. Plotted values on each graph are copy number means of technical triplicates calculated for each sample relative to the 0% control ± SEM. Related to Figures 5 and 6. (B) The sensitivity of the ddPCR assay on the RainDrop digital PCR platform. The ddPCR analysis of samples with varying ratios of abnormal cells harbouring 17q trisomy. Plotted are copy number values for two biological replicates.

Supplemental Tables

Table S1. Statistically-determined expected numbers of abnormal cells for different sample sizes (n) at given levels of mosaicism; related to Figures 2, 3, S1-S4. Each row represents a level of mosaicism (1-90%). The columns have lower (ll) and upper (ul) limits on the expected number of cells detected at 95% confidence for different sample sizes (n).

	**n=5**		**n=10**		**n=20**		**n=30**		**n=40**		**n=45**		**n=50**		**n=60**		**n=80**		**n=100**	
	**ll**	**ul**	**ll**	**ul**	**ll**	**ul**	**ll**	**ul**	**ll**	**ul**	**ll**	**ul**	**ll**	**ul**	**ll**	**ul**	**ll**	**ul**	**ll**	**ul**
**1%**	0	1	0	1	0	1	0	2	0	2	0	2	0	2	0	2	0	3	0	3
**2%**	0	1	0	1	0	2	0	2	0	3	0	3	0	3	0	4	0	4	0	5
**6%**	0	2	0	2	0	4	0	5	0	6	0	6	0	7	1	8	1	9	2	11
**9%**	0	2	0	3	0	5	0	6	1	7	1	8	1	9	2	10	3	13	4	15
**10%**	0	2	0	3	0	5	0	7	1	8	1	9	1	9	2	11	3	14	5	16
**13%**	0	2	0	4	0	6	1	8	1	10	2	11	2	11	3	13	5	17	7	20
**18%**	0	3	0	4	1	7	2	10	3	12	3	13	4	15	5	17	8	21	11	26
**19%**	0	3	0	5	1	7	2	10	3	13	4	14	4	15	6	18	9	22	12	27
**24%**	0	3	0	5	1	9	3	12	5	15	5	17	6	18	8	21	12	27	16	33
**28%**	0	3	0	6	2	10	4	13	6	17	7	19	8	20	10	24	15	30	19	37
**61%**	1	5	3	9	8	16	13	23	18	30	21	34	24	37	29	44	40	57	51	70
**77%**	2	5	5	10	12	19	18	27	25	36	29	40	32	44	40	52	54	69	69	85
**90%**	3	5	7	10	15	20	23	30	32	39	36	44	41	49	49	58	66	77	84	95

Table S2. Karyotypes of the cells used in the qPCR assays; related to Figure 4. For some of the cell lines the FISH analysis with a gene-specific DNA probe for the *BCL2L1* gene was performed in parallel with the qPCR and G-banding.

HPSC line	Passage number	Karyotype [number of metaphases scored]	% cells with three copies of 20q11.21 as determined by FISH
H7.s14	2+4+4+3+14	46,XX[30]	4%
Shef6 8H12	14+43+5+3+3+5	46,XX[30]	1%
Shef5	12+14+5+4+4	46,XX[20]	Not tested
MasterShef 8	21	46,XY[20]	8%
MasterShef 14	16	46,XX[30]	8%
H14.s9	9+6	46,XY[30]	Not tested
H7.s14-Tomato	23+3+10+6	46,XX[30]	14%
H14BJ1	92+11+6+5+6	48,XY,+12,+17,der(17)del(17)(p13)hsr(17)(p11.2)[10]	Not tested
Shef5-SF9	22+15+3+30+3+5+7	47,XX,t(1;11),trp(17)(p11.2),+20[20]	100%
H7.s6	62+33+2+4+34+5	47,XX,+del(1)(p22p22),der(6)t(6;17)(q27;q1)[10]	Not tested
HES3-MIXL	50+8/27+7+4+5+7	46,XX,add(10)(q24)[2]/ 46,XX[28]	53%
Shef6 2A7	14+43+5+3+3+6	46,XX[30]	41%

SUPPLEMENTAL EXPERIMENTAL PROCEDURES

The human pluripotent stem cell lines

H7 and H14 lines (Thomson et al. (1998)) were a gift from Dr James Thomson, University of Wisconsin. H7.s14, H7.s6, H14.s9 and H14BJ1 sublines were established at the University of Sheffield (Draper et al. (2004), Enver et al. (2005)). H7.s14-Tomato subline is stably expressing tdTomato fluorescent protein, and H7.s6-GFP and H14BJ1-GFP are stably expressing green fluorescent protein (GFP). MasterShef 8 and MasterShef 14 hPSC lines were derived to Good Manufacturing Practice Conditions at the University of Sheffield. Shef5 was derived at the University of Sheffield (Aflatoonian et al., 2010), and Shef5-SF9 subline was established at the University of Sheffield from the original Shef5 line. Shef6 8H12 and Shef6 2A7 are sublines of Shef6 hPSC line (Aflatoonian et al., 2010) derived at the University of Sheffield. HES3-MIXL is a HES3 line with a GFP knocked into the *MIXL1* allele obtained from A. Elefanty and E. Stanley (Davis et al., 2008).

Mixing experiments for detecting the sensitivity of karyotyping

H7.s14 or H7.s14-Tomato cells grown in mTESR medium (STEMCELL Technologies, Vancouver, Canada) on matrigel (BD Biosciences, Oxford, UK) were treated with collagenase IV, washed in mTESR and scraped off the flask to obtain small clumps of cells. The clumps were plated into 6 well plates (Corning Costar, High Wycombe, UK) on matrigel in mTESR and left for 48h. Cells from a spare well were counted to determine the cell numbers per well. H7.s6-GFP cells grown on matrigel in mTESR were dissociated into single cells using TrypLE (Life Technologies). Dissociated cells were washed with mTESR and counted. This was followed by plating of increasing numbers of dissociated H7.s6-GFP cells onto H7.s14 or H7.s14-Tomato cells grown in colonies to achieve the following ratios of

H7.s6-GFP cells in the H7.s14 or H7.s14-Tomato cultures: 1%, 6%, 9%, 13%, 18% and 28%. Mixed cultures were left for 24h to ensure that plated cells recover and restart cycling. The mixed cultures of H7.s14 and H7.s6-GFP cells were then stained with Hoechst 33342 and imaged on the InCell Analyser to confirm the ratios of normal and variant cells. The images were analysed using the Developer Toolbox 1.7 software (GE Healthcare) to determine the number of GFP expressing cells and the total number of nuclei based on Hoechst33342 staining. The ratios of abnormal cells were calculated as the number of GFP-expressing cells divided by the total number of nuclei per well. In the case of H7.s14-Tomato and H7.s6-GFP cells, the mixed cultures were imaged on the InCell Analyser for Tomato and GFP expression (without staining for Hoechst 33342) to confirm the ratios of cells plated. The images were analysed using the Developer Toolbox 1.7 software (GE Healthcare) to determine the number of GFP and tdTomato- expressing cells. The ratios of abnormal cells were determined as the number of GFP-expressing cells divided by the sum of the GFP-expressing and tdTomato-expressing cells. Following the imaging, mosaic cultures were treated with colcemid and processed for G-banding, as detailed below.

As an alternative way of constructing mosaic samples and determining the ratios of abnormal cells present in cultures, diploid cells (H7.s14 or H14.s9) and their GFP-expressing abnormal counterparts (H7.s6-GFP or H14BJ1-GFP) were dissociated into single cells using Single Cell Dissociation solution (Sigma-Aldrich, Poole, Dorset, UK) and counted. Different ratios of diploid and aneuploid cells were plated into flasks on vitronectin in Essential 8 and left to grow for 4-5 days. The flasks were then passaged at 1:2 ratio using ReLeSR (STEMCELL Technologies) and scraping, ensuring that all the cells are harvested from the flask. Each sample was passaged into two flasks on vitronectin in Essential 8. For H14 mixtures, the 10μM Y-27632 inhibitor (Sigma-Aldrich) was included in the media at the time of plating for 24h to aid with the survival of diploid cells. Two days post-plating, the cells

from one of the flasks were harvested using trypsin, ensuring all the cells are collected from the flask. After washing the cells in PBS supplemented with 10% fetal calf serum, the samples were analysed for the presence of GFP-labeled variant cells by flow cytometry using BD FACSJazz (BD Biosciences, Oxford, UK). H7.s14 or H14.s9 control samples were used to determine the gating threshold for GFP fluorescence and H7.s6-GFP or H14BJ1-GFP cells were used as a positive control for gating. At the same time, the duplicate flask was colcemid-treated and processed for G-banding, as detailed below. After the preparation of metaphase spreads on glass microscope slides, slides were scanned, metaphase images captured and analysed using the Leica Biosystems Cytovision Image Analysis system (version 7.3.2 build 35). The metaphase cells, having been thoroughly mixed in suspension during harvesting and slide making, were sorted based upon the location on the slide, achieving a random selection of cells for scoring. Metaphase cells were scored for the presence or absence of the abnormal chromosome 6, der(6)t(6;17)(q27;q1) in the H7.s14 and H7.s6-GFP mixed samples, or for the presence or absence of an additional abnormal chromosome 17, der(17)(del(17)(p13.3)hsr(17)(p11.2) in the H14.s9 and H14.BJ-GFP mixed samples. The standard number of metaphase cells used for a mosaic screen in clinical cytogenetics ranges from 30-45 (ACGS Best Practice Guidelines, http://www.acgs.uk.com) and therefore the number of cells scored here ranged from 5 to 100. Each metaphase cell was scored once only. Those cells in which the region of interest was unscorable (those where the chromosome was obscured, broken or missing) were excluded from analysis.

G-banding

Cells were treated with 0.1 µg/ml colcemid (Invitrogen) for up to 4h. After dissociation with 0.25% trypsin/versene (Gibco, Invitrogen), the cells were re-suspended in pre-warmed 0.0375M KCl hypotonic solution and incubated for 10min at room temperature. Cells were

then pelleted and resuspended in fixative (3:1 methanol:acetic acid). Metaphase spreads were prepared on glass microscope slides and G-banded by brief exposure to trypsin and stained with 4:1 Gurr's/Leishmann's stain (Sigma). Slides were scanned, metaphase images captured and analysed using the Leica Biosystems Cytovision Image Analysis system (version 7.3.2 build 35).

Statistically-determined expected numbers of abnormal cells for different sample sizes at given levels of mosaicism

A custom R code was implemented to calculate the upper and lower limits of the numbers of abnormal cells that would be expected when sampling a given number of cells, based on the confidence levels indicated in Hook et al. (1977).

DNA extraction and restriction digest

DNA from hPSCs was extracted using the Blood and Tissue Kit for DNA Extraction (QIAGEN, Crawley, UK). DNA was quantified by absorption spectroscopy (NanoPhotometer, Implen, Munich, Germany). Restriction digestion of DNA was performed in 100µl reactions containing 1µg of DNA and 10 units of *EcoRI* enzyme (Fermentas, ThermoFisher Scientific) in 1X *EcoRI* buffer (Fermentas). Reactions were incubated for 2h at 37°C and the enzyme was then inactivated by incubating at 65°C for 20min.

Primer design and validation

The genomic sequences of genes were obtained from the Ensembl Genome Browser Database. Gene-specific primers and the appropriate hydrolysis probes were designed in the intronic region of genes by the Universal Probe Library (UPL) Assay Design Centre web facility (Roche Diagnostics, Mannheim, Germany)

(http://lifescience.roche.com/shop/en/us/overviews/brand/universal-probe-library). NCBI Primer-BLAST was used to check primer specificity *in silico*. Newly designed primers were tested empirically using melting curve analysis. For this, 20µl PCR reactions were prepared in 384-well plates containing 1x SYBR Green JumpStart Taq Ready Master Mix (SigmaAldrich), 100nM of each of the forward and reverse primers, and 20ng of *EcoRI*-digested DNA template. Reactions were run on the QuantStudio 12K Flex Real-Time PCR System (Applied Biosystems, Life Technologies). Samples were heated to 50°C for 2min and denatured at 95°C for 10min. This was followed by 40 cycles of 95°C for 15s and 60°C for 1min. Melting curve analysis was performed by heating the samples to 95°C for 15s. The samples were then cooled and held at 60°C for 1min. This was followed by heating the samples to 95°C at a rate of 0.05°C/s under continuous fluorescence monitoring. The melting curves were obtained using the QuantStudio 12K Flex Software (Applied Biosystems, Life Technologies).

Primer amplification efficiencies for each set of primers were calculated by preparing a 5-fold dilution series of gDNA (100, 20, 4, 0.8 and 0.16ng) in triplicate or quadruplicate wells and plotting the average Cq values of replicates against the input DNA quantity in log10 scale. The data was fit to a straight line and the slope of the line was calculated. The primer efficiency was calculated as $-1+10^{(-1/slope)}$. Primers with efficiencies between 90% and 115% were used for the target and reference genes (**Table 1**).

Calculation of copy numbers of target genes relative to the calibrator samples in the qPCR assay

The Cq values obtained from the QuantStudio 12K Flex Software with auto baseline settings and were exported to Excel for copy number analysis using the relative quantification method (2^{-ddcq}) as follows:

[1] dCq(target gene) = Cq(target gene) – average Cq (reference gene, *RELL1*)

[2] ddCq1= dCq(target gene of the sample) – average dCq (target gene of the calibrator 1)

[3] Relative quantity of the target gene in the sample compared to calibrator 1 (RQ1) = 2^{-ddCq1}

[4] Copy number relative to calibrator 1 (CN1) = Number of copies of the target gene in the calibrator * RQ1 = 2* RQ1

[5] ddCq2= dCq(target gene of the sample) – average dCq (target gene of the calibrator 2)

[6] Relative quantity of the target gene in the sample compared to calibrator 2 (RQ2) = 2^{-ddCq2}

[7] Copy number relative to calibrator 2 (CN2) = Number of copies of the target gene in the calibrator * RQ2 = 2* RQ2

[8] Average copy number relative to both calibrators = Average of the CN1 and CN2

Digital droplet PCR using the RainDance PCR platform

Reaction mixtures (25µL) contained 1x TaqMan Genotyping Master Mix (ThermoFisher Scientific), 225nM of each primer and 50nM of each probe (TaqMan assays for *TK1* (FAM-labelled) and *TERT* (reference, VIC-labelled*)* (Life Technologies)), 1x dPCR Droplet Stabiliser (Raindance Technologies, Billerica, MA) and 150 ng DNA (as quantified by NanoPhotometer, Implen, Munich, Germany). Droplets were formed in the RainDrop source instrument (RainDance Technologies). The DNA was amplified in a thermal cycler using the following conditions: 95°C for 10min, followed by 45 cycles of 95°C for 15s and 60°C for 1min. The final extension step was at 98°C for 10min before the reactions were cooled down to 12°C and transferred into RainDrop Sense chip (RainDance Technologies) for fluorescence measurements of the probes (FAM and VIC). The data was analysed using the RainDrop Analyst Software (RainDance Technologies).

Supplemental Information for Figure 1:

Data of karyotypic abnormalities in human embryonic stem cells is based on the following references: (Amps et al., 2011; Baker et al., 2007; Brimble et al., 2004; Buzzard et al., 2004; Caisander et al., 2006; Catalina et al., 2008; Cowan et al., 2004; Draper et al., 2004; Gertow et al., 2007; Hasegawa et al., 2006; Herszfeld et al., 2006; Imreh et al., 2006; Inzunza et al., 2004; Lagarkova et al., 2008; Lefort et al., 2008; Ludwig et al., 2006; Maitra et al., 2005; Mitalipova et al., 2005; Narva et al., 2010; Spits et al., 2008; Ware et al., 2006).

Supplemental References

Aflatoonian, B., Ruban, L., Shamsuddin, S., Baker, D., Andrews, P., and Moore, H. (2010). Generation of Sheffield (Shef) human embryonic stem cell lines using a microdrop culture system. In Vitro Cell Dev Biol Anim *46*, 236-241.

Amps, K., Andrews, P.W., Anyfantis, G., Armstrong, L., Avery, S., Baharvand, H., Baker, J., Baker, D., Munoz, M.B., Beil, S., *et al.* (2011). Screening ethnically diverse human embryonic stem cells identifies a chromosome 20 minimal amplicon conferring growth advantage. Nat Biotechnol *29*, 1132-1144.

Baker, D.E., Harrison, N.J., Maltby, E., Smith, K., Moore, H.D., Shaw, P.J., Heath, P.R., Holden, H., and Andrews, P.W. (2007). Adaptation to culture of human embryonic stem cells and oncogenesis in vivo. Nat Biotechnol *25*, 207-215.

Brimble, S.N., Zeng, X., Weiler, D.A., Luo, Y., Liu, Y., Lyons, I.G., Freed, W.J., Robins, A.J., Rao, M.S., and Schulz, T.C. (2004). Karyotypic stability, genotyping, differentiation, feeder-free maintenance, and gene expression sampling in three human embryonic stem cell lines derived prior to August 9, 2001. Stem Cells Dev *13*, 585-597.

Buzzard, J.J., Gough, N.M., Crook, J.M., and Colman, A. (2004). Karyotype of human ES cells during extended culture. Nat Biotechnol *22*, 381-382; author reply 382.

Caisander, G., Park, H., Frej, K., Lindqvist, J., Bergh, C., Lundin, K., and Hanson, C. (2006). Chromosomal integrity maintained in five human embryonic stem cell lines after prolonged in vitro culture. Chromosome Res *14*, 131-137.

Catalina, P., Montes, R., Ligero, G., Sanchez, L., de la Cueva, T., Bueno, C., Leone, P.E., and Menendez, P. (2008). Human ESCs predisposition to karyotypic instability: Is a matter of culture adaptation or differential vulnerability among hESC lines due to inherent properties? Mol Cancer *7*, 76.

Cowan, C.A., Klimanskaya, I., McMahon, J., Atienza, J., Witmyer, J., Zucker, J.P., Wang, S., Morton, C.C., McMahon, A.P., Powers, D., *et al.* (2004). Derivation of embryonic stem-cell lines from human blastocysts. N Engl J Med *350*, 1353-1356.

Davis, R.P., Ng, E.S., Costa, M., Mossman, A.K., Sourris, K., Elefanty, A.G., and Stanley, E.G. (2008). Targeting a GFP reporter gene to the MIXL1 locus of human embryonic stem cells identifies human primitive streak-like cells and enables isolation of primitive hematopoietic precursors. Blood *111*, 1876-1884.

Draper, J.S., Smith, K., Gokhale, P., Moore, H.D., Maltby, E., Johnson, J., Meisner, L., Zwaka, T.P., Thomson, J.A., and Andrews, P.W. (2004). Recurrent gain of chromosomes 17q and 12 in cultured human embryonic stem cells. Nat Biotechnol *22*, 53-54.

Gertow, K., Cedervall, J., Unger, C., Szoke, K., Blennow, E., Imreh, M.P., and Ahrlund-Richter, L. (2007). Trisomy 12 in HESC leads to no selective in vivo growth advantage in teratomas, but induces an increased abundance of renal development. J Cell Biochem *100*, 1518-1525.

Hasegawa, K., Fujioka, T., Nakamura, Y., Nakatsuji, N., and Suemori, H. (2006). A method for the selection of human embryonic stem cell sublines with high replating efficiency after single-cell dissociation. Stem Cells *24*, 2649-2660.

Herszfeld, D., Wolvetang, E., Langton-Bunker, E., Chung, T.L., Filipczyk, A.A., Houssami, S., Jamshidi, P., Koh, K., Laslett, A.L., Michalska, A.*, et al.* (2006). CD30 is a survival factor and a biomarker for transformed human pluripotent stem cells. Nat Biotechnol *24*, 351-357.

Imreh, M.P., Gertow, K., Cedervall, J., Unger, C., Holmberg, K., Szoke, K., Csoregh, L., Fried, G., Dilber, S., Blennow, E.*, et al.* (2006). In vitro culture conditions favoring selection of chromosomal abnormalities in human ES cells. J Cell Biochem *99*, 508-516.

Inzunza, J., Sahlen, S., Holmberg, K., Stromberg, A.M., Teerijoki, H., Blennow, E., Hovatta, O., and Malmgren, H. (2004). Comparative genomic hybridization and karyotyping of human embryonic stem cells reveals the occurrence of an isodicentric X chromosome after long-term cultivation. Mol Hum Reprod *10*, 461-466.

Lagarkova, M.A., Volchkov, P.Y., Philonenko, E.S., Pfannkuche, K., Prokhorovich, M.A., Zabotina, T., Hescheler, J., and Kiselev, S.L. (2008). CD 30 is a marker of undifferentiated human embryonic stem cells rather than a biomarker of transformed hESCs. Cell Cycle *7*, 3610-3612.

Lefort, N., Feyeux, M., Bas, C., Feraud, O., Bennaceur-Griscelli, A., Tachdjian, G., Peschanski, M., and Perrier, A.L. (2008). Human embryonic stem cells reveal recurrent genomic instability at 20q11.21. Nat Biotechnol *26*, 1364-1366.

Ludwig, T.E., Levenstein, M.E., Jones, J.M., Berggren, W.T., Mitchen, E.R., Frane, J.L., Crandall, L.J., Daigh, C.A., Conard, K.R., Piekarczyk, M.S.*, et al.* (2006). Derivation of human embryonic stem cells in defined conditions. Nat Biotechnol *24*, 185-187.

Maitra, A., Arking, D.E., Shivapurkar, N., Ikeda, M., Stastny, V., Kassauei, K., Sui, G., Cutler, D.J., Liu, Y., Brimble, S.N.*, et al.* (2005). Genomic alterations in cultured human embryonic stem cells. Nat Genet *37*, 1099-1103.

Mitalipova, M.M., Rao, R.R., Hoyer, D.M., Johnson, J.A., Meisner, L.F., Jones, K.L., Dalton, S., and Stice, S.L. (2005). Preserving the genetic integrity of human embryonic stem cells. Nat Biotech *23*, 19-20.

Narva, E., Autio, R., Rahkonen, N., Kong, L., Harrison, N., Kitsberg, D., Borghese, L., Itskovitz-Eldor, J., Rasool, O., Dvorak, P.*, et al.* (2010). High-resolution DNA analysis of human embryonic stem cell lines reveals culture-induced copy number changes and loss of heterozygosity. Nat Biotechnol *28*, 371-377.

Spits, C., Mateizel, I., Geens, M., Mertzanidou, A., Staessen, C., Vandeskelde, Y., Van der Elst, J., Liebaers, I., and Sermon, K. (2008). Recurrent chromosomal abnormalities in human embryonic stem cells. Nat Biotechnol *26*, 1361-1363.

Ware, C.B., Nelson, A.M., and Blau, C.A. (2006). A comparison of NIH-approved human ESC lines. Stem Cells *24*, 2677-2684.
